# Interplay of Structural Disorder and Short Binding Elements in the Cellular Chaperone Function of Plant Dehydrin ERD14

**DOI:** 10.3390/cells9081856

**Published:** 2020-08-07

**Authors:** Nikoletta Murvai, Lajos Kalmar, Bianka Szalaine Agoston, Beata Szabo, Agnes Tantos, Gyorgy Csikos, András Micsonai, József Kardos, Didier Vertommen, Phuong N. Nguyen, Nevena Hristozova, Andras Lang, Denes Kovacs, Laszlo Buday, Kyou-Hoon Han, Andras Perczel, Peter Tompa

**Affiliations:** 1 Institute of Enzymology, Research Centre for Natural Sciences, 1117 Budapest, Hungary; murvai.nikoletta@ttk.hu (N.M.); lk397@cam.ac.uk (L.K.); szalaineagostonbianka@gmail.com (B.S.A.); szabo.beata@ttk.mta.hu (B.S.); tantos.agnes@ttk.mta.hu (A.T.); buday.laszlo@ttk.mta.hu (L.B.); 2 Department of Veterinary Medicine, University of Cambridge, Cambridge CB3 0ES, UK; 3 MTA-ELTE Protein Modelling Research Group and Laboratory of Structural Chemistry and Biology, Institute of Chemistry, Eötvös L. University, 1117 Budapest, Hungary; langax@chem.elte.hu (A.L.); perczel.andras@ttk.elte.hu (A.P.); 4 Department of General Zoology, Eötvös Loránd University, 1117 Budapest, Hungary; gcsikos@elte.hu; 5 ELTE NAP Neuroimmunology Research Group, Department of Biochemistry, Institute of Biology, Eötvös Loránd University, 1117 Budapest, Hungary; micsonai@ttk.elte.hu (A.M.); kardos@elte.hu (J.K.); 6 Faculty of Medicine and de Duve Institute, Université Catholique de Louvain, 1200 Brussels, Belgium; didier.vertommen@uclouvain.be; 7 Structural Biology Brussels (SBB), Vrije Universiteit Brussel (VUB), 1050 Brussels, Belgium; Phuong.Nguyen.Nhu@vub.be (P.N.N.); hristozova.nevena@gmail.com (N.H.); denes.s.kovacs@gmail.com (D.K.); 8 VIB-VUB Center for Structural Biology (CSB), Vlaams Instituut voor Biotechnologie (VIB), 1050 Brussels, Belgium; 9 Gene Editing Research Center, Division of Convergent Biomedical Research, Korea Research Institute of Bioscience and Biotechnology, Daejeon 34141, Korea; yoonje55@naver.com; 10 Biomedical Translational Research Center, Division of Convergent Biomedical Research, Korea Research Institute of Bioscience and Biotechnology, Daejeon 34141, Korea

**Keywords:** intrinsic structural disorder, chaperone, in-cell NMR, pre-structured motif, client protein, cell protection

## Abstract

Details of the functional mechanisms of intrinsically disordered proteins (IDPs) in living cells is an area not frequently investigated. Here, we dissect the molecular mechanism of action of an IDP in cells by detailed structural analyses based on an in-cell nuclear magnetic resonance experiment. We show that the ID stress protein (IDSP) *A. thaliana* Early Response to Dehydration (ERD14) is capable of protecting *E. coli* cells under heat stress. The overexpression of ERD14 increases the viability of *E. coli* cells from 38.9% to 73.9% following heat stress (50 °C × 15 min). We also provide evidence that the protection is mainly achieved by protecting the proteome of the cells. In-cell NMR experiments performed in *E. coli* cells show that the protective activity is associated with a largely disordered structural state with conserved, short sequence motifs (K- and H-segments), which transiently sample helical conformations in vitro and engage in partner binding in vivo. Other regions of the protein, such as its S segment and its regions linking and flanking the binding motifs, remain unbound and disordered in the cell. Our data suggest that the cellular function of ERD14 is compatible with its residual structural disorder in vivo.

## 1. Introduction

Intrinsically disordered proteins and regions (IDPs/IDRs) exist, and apparently function, without a well-defined structure [1,2]. The functioning of IDPs/IDRs is thought to rely on two distinct mechanistic elements: (1) molecular recognition, when they bind partner molecule(s) in an induced folding process, and (2) entropic chain function, when their function stems directly from the disordered state [3]. Whereas these mechanisms have been observed in many cases in vitro, their contribution to function and their possible interplay have hardly ever been analyzed under cellular, truly in vivo conditions.

We suggest here that the cellular observation of the functioning of IDP chaperones may provide a unique opportunity to observe the atomic details of the functioning of IDPs in situ. Alongside traditional chaperones, such as Hsp90 and GroEL/GroES [4,5], recent evidence points to the existence of several IDPs/IDRs of potent chaperone activity, at least in an in vitro situation [6,7,8,9]. For example, late embryogenesis abundant (LEA) proteins of plants (e.g., Early Response to Dehydration (ERD14) of *A. thaliana* [10]) or animals (e.g., AavLEA of the desiccation-tolerant organism *A. avenae* [11]), or other unrelated IDPs, e.g., bacterial Hsp33 [12] and tardigrade dehydrins [13], protect other proteins against denaturation caused by elevated temperatures or dehydration. This phenomenon, primarily associated with group 2 LEA proteins (dehydrins) [14,15] and hydrophilins [16,17], may represent a novel, little understood layer of cellular protection in living organisms by ID stress proteins (IDSPs). IDSPs apparently use their structural plasticity in client protection [9,18], in contrast to globular chaperones, where a 3D fold is required for function [5].

Dehydrins are encoded by 10 genes in *A. thaliana* [6,19] and may fulfill overlapping functions. Most of them are largely disordered in vitro [7,8], and they are classified by the presence and pattern of short conserved motifs such as (1) Lys-rich K-segments, (2) a region showing homology with classical chaperones (ChP-segment), (3) an oligo-Ser repeat (S-segment), and (4) a slightly hydrophobic region (H region), which appear in different combinations in different dehydrins [8,18]. The rather broad and non-specific chaperone activity of dehydrins and other LEA proteins has been demonstrated by a range of non-physiological substrates [7,20,21] and protein mixtures [21,22] in vitro. They also exert a general protective effect in heterologous cellular systems, i.e., when overexpressed in *E. coli* [21,23,24], yeast [20,25], plant [21,26], or human [22,27] cells. Their cellular protective effect has been observed in a variety of stress conditions, such as high temperature, freezing, osmotic shock, or high salinity, whereas in vitro, they have been observed to preserve the activity of enzymes [7,20,21,28], or to prevent the aggregation of proteins [21,22]. Although in reconstituted systems they have been shown to protect a broad range of test proteins, their physiological partners have not yet been identified. In terms of their molecular mechanism(s) of action, only general schemes, such as molecular shielding, space filling, membrane stabilization, or entropy transfer, have been suggested [7,9]. Plant stress is also averted by the cellular synthesis of small-molecule natural compounds (e.g., proline), which favor the folding of proteins and thus protect them from denaturation [29]. However, their effective concentration is usually in the molar range; thus, the chaperone-like effect of dehydrins is not accounted for by this model. ERD14, which is highly overexpressed in *A. thaliana* cells upon heat and dehydration stress [6,30], is a perfect model protein to study if we want to decide which of the above-mentioned molecular mechanisms is compatible with physiological observations. It is a potent chaperone with a variety of substrates in vitro [10], and its K-segments sample secondary structural states [31] in the largely disordered structural ensemble of the whole protein. It is probably of high relevance that induced the folding of short, pre-formed recognition motifs or pre-structured motifs (PreSMos) is a general theme in IDP function in vitro [32,33], with limited in vivo evidence coming from a few in-cell NMR studies [34,35,36,37]. Here, we overexpressed ERD14 in *E. coli* cells, which enables us to conduct in-cell NMR measurements to uncover the structural mechanism of an IDSP in vivo. We found that the functionality of ERD14 is linked with its locally pre-structured motifs that engage in binding to partner molecules, and disordered regions that solvate and hold partners apart, providing a direct link between structural disorder and protein function in live cells.

## 2. Materials and Methods

### 2.1. Constructs

Wild-type ERD14 was expressed from a previously prepared construct of ERD14 in a pET22b vector [30], which was cloned to contain no additional amino acids next to the original ERD14 sequence. The pelB leader sequence was eliminated by introducing a stop codon before its sequence in the vector to ensure cytoplasmic localization of the protein.

For Full-Scambled (Full-Scr) ERD14, amino acids except for the starting methionine were scrambled in silico. The resulting amino acid sequence was checked for the lack of coils, secondary structures, and overall folding tendency to make sure that the protein possessed a truly disordered nature. The final sequence was chosen from over 100 different variants, and the coding sequence was generated by full cDNA synthesis. The construct was cloned into a pET22b vector so that it did not contain any additional amino acids other than the scrambled sequence indicated in Table S1 (Supplementary Materials). The pelB leader sequence was removed the same way as in the case of the wild-type variant.

For in-cell half-life measurements and quantification, the DNA fragment coding wild-type ERD14 was amplified by Pfu Turbo HotStart DNA polymerase (Agilent, Santa Clara, CA, USA) by PCR using ccgctcgagtgATGGCTGAGGAAATCAAGAATGTTCCTG forward and gaagatctTTCTTTATCTTTCTTCTCCTCCTCTACGG reverse primers and subcloned into the XhoI/BglII sites of expression vector pT7-FLAG 2 (Sigma, St. Louis, MO, USA). The C-terminal FLAG tagged fusion protein was expressed in the cytoplasm of BL21 star (DE3) cells under the control of the T7/lac promoter. The pGEX-5X 1 vector was used to express glutathione S-transferase (GST) or calpastatin protein in the same cell line as a control.

### 2.2. E. Coli Cell Viability Assays

**Figure 1:**
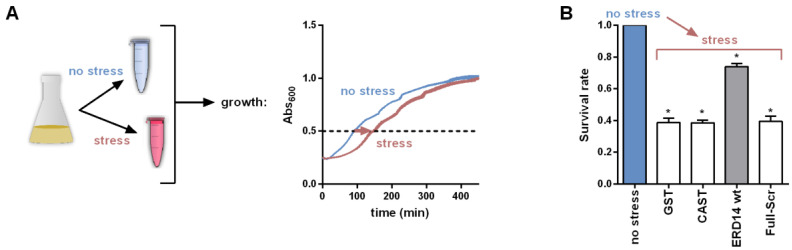
Protection of *E. coli* cells against stress by Early Response to Dehydration (ERD14). (**A**) Scheme of the cell viability assay. After a standard induction period, cells are stressed by a heat shock of 50 °C × 15 min. Viability of stressed and non-stressed samples were compared based on their cell growth after appropriate dilution into fresh medium. (**B**) Survival rate (viability) is reduced to about 39% (compared to non-stressed cells) in the case of overexpression of control proteins (glutathione S-transferase (GST) and intrinsically disordered proteins (IDP) Calpastatin (CAST)), whereas the overexpression of ERD14 increases the survival rate to about 73.9% after stress, which is a highly significant difference. Keeping the amino acid composition but scrambling the sequence of ERD14 (Full-Scr ERD14) completely abolishes its protective effect. Survival rates are shown as mean values with SEM, significant differences are labeled with asterisks (*).

The viability of *E. coli* cells was measured in LB medium, by observing the time it takes for cells to reach an optical density of 0.55 at 600 nm of appropriately diluted samples. To this end, BL21 Star (DE3) *E. coli* cells overexpressing WT or Full-Scr ERD14 or control proteins (GST, calpastatin (CAST)) were grown in 5 mL LB medium containing 50 µg/mL carbenicillin overnight in 37 °C under continuous shaking at 200 rpm. The next day, 50 µL aliquots were transferred into 5 mL LB medium containing 50 µg/mL carbenicillin and were grown for 2 h. Protein expression was induced with 0.5 mM IPTG for 3 h. Then, 0.5 mL aliquots were taken and either stressed or not stressed before appropriate dilution into LB medium into the wells of a 96-well plate. Measurements of absorbance at 600 nm were taken every 2 min in a BioTek Synergy Mx microplate reader for 12 h at 37 °C with 200 rpm continous shaking: viability was associated with the time taken for the cells to reach an optical density of 0.55, which was roughly the half-maximal value they attain after long incubation periods (Figure 1A). From a number of different conditions, we selected 50 °C × 15 min for an optimal stress effect. The observed protection effect was normalized to the actual expression level of the given construct in the given experiment. In short, the actual concentration was determined, and the expected protection at a concentration matching that of ERD14WT was calculated by linear intra- or extrapolation. Further details, such as optimization of the assay, are given in the Supplementary Information.

### 2.3. In-Cell NMR and Spectral Assignment

In-cell NMR was measured on *E. coli* cells in which ERD14 was overexpressed under ^15^N- or ^15^N- and ^13^C- labeling conditions. To this end, BL21 Star (DE3) pLysS cells were transformed with wild-type ERD14 protein or Full-Scr ERD14, grown in LB medium, and transfered one hour prior to induction to 0.25 volume of M9 minimal medium complemented with ^15^N-labeled ammonium chloride. For the assignment both ^15^N-labeled ammonium chloride and ^13^C-labeled glucose were added. Finally, ERD14 expression was induced by 1.6 mM IPTG for 3 h at 37 °C. For the NMR measurement, 100 mL of cell culture were pelleted by gentle centrifugation and resuspended in 800 µL of ^15^N-free M9 minimal medium (NMR-M9). Then, 1 mL of this dense cell suspension was complemented with 10% D2O and placed into a standard 5 mm NMR tube. ^15^N-HSQC spectra were recorded on a Bruker DRX 500 MHz spectrometer at 277 K. Evaluation of the observed in-cell spectrum was performed by comparison with the appropriate in vitro reference spectra of purified ERD14 WT and Full-Scr ERD14 recorded under a variety of conditions (NMR-M9, pH 6.6–7.7, temperature 277–280 K, dextran concentration 0–400 mg/mL, Figures S1 and S2 (Supplementary Materials)). The previously performed assignment (at pH 6.5, 288 K, BMRB entry 16,876 for wt ERD14) was transferred along this series of in vitro HSQC spectra to conditions similar to the in-cell spectra, i.e., 277 K and pH 7.7–7.9 within the *E. coli* cell (BMRB Accession Number 26636). To prepare lysates of in-cell NMR samples, cells were sedimented for 6 min at 3000× *g* and resuspended in NMR-M9 buffer. After sonication and boiling for 5 min, the denatured globular proteins were sedimented by centrifugation for 30 min at 12,100 rpm. The cell extract was purified on ResourceQ anion exchange column, dialyzed into MQ water, and lyophilized. The purified protein was dissolved in NMR-M9 buffer and transferred to the original NMR tubes. Then, 1D/2D NMR spectra were recorded with an identical spectrometer and acquisition settings as for the intact in-cell NMR samples. The quantity and intactness of ERD14 WT and Full-Scr were analyzed on SDS-PAGE gel. Comparing the obtained peak intensity ratios and 1D signal ratios of in-cell and purified protein NMR spectra and furthermore the SDS-PAGE gels, the protein concentration in the cell was around 177 ± 30 µM. To characterize regions involved in partner binding via severe line broadening or the disappearance of selected resonances of the in-cell HSQC spectra compared to the reference, we analyzed assigned peaks and identified “steady”, “disappearing”, and “broadening” peaks (Table S2 (Supplementary Materials)). Further details and control measurements can be found in the Supplementary Information.

### 2.4. Protein Stability in the Cell

To demonstrate the intracellular stability of ERD14, BL21 Star (DE3) cells were transformed with ERD14/pT7-FLAG 2 construct. A moderate expression of FLAG-ERD14 was induced by 0.5 mM IPTG in order to avoid overloading the cellular systems, and cells were either kept under normal or stressed conditions. ERD14 expression was arrested by the removal of IPTG by washing the cells. FLAG-ERD14 was visualized by monoclonal Anti-FLAG M2 antibody. Further details are found in the Supplementary Information.

### 2.5. Pull-Down and Mass-Spectrometric Analysis of Cellular ERD14 Partners

Wild-type (WT) ERD14 was subcloned into pAN4 vector (Avidity) and transformed into AVB101 bacterial strain for in-cell biotinylation. Expression and biotinylation were performed according to the manufacturer’s recommendation. Cells were collected and washed 3 times with PBS, and cross-linked with dithiobis (succinimidyl propionate) (DSP) (Lomant’s reagent) at 2 mM concentration at 30 °C for 7 min. After lysis, biotin–ERD14–substrate complexes were pulled down with Streptavidin–MAG–Sepharose (GE Healthcare Bio-Sciences AB, Uppsala, Sweden) according to the manufacturer’s protocol. Samples were run on SDS-PAGE, in-gel digested by trypsin, and analyzed by LC-MS/MS on an ESI-IT LTQ XL mass-spectrometer (Thermo Fisher Scientific, Waltham, MA, USA). The resulting MS/MS spectra were searched using the algorithm SequestHT against a target-decoy Escherichia Coli Protein database obtained from Uniprot. Peptide matches were filtered using the q-value and Posterior Error Probability calculated by the Percolator algorithm ensuring an estimated false positive rate below 5%. The filtered SEQUEST HT output files for each peptide were grouped according to the protein from which they were derived using the multiconsensus results tool within Proteome Discoverer 1.4, and gel slices at the same molecular weight of stressed and non-stressed samples were compared to each other. The background of the pulldown experiment has been determined in an identical experiment with non-biotinylated ERD14.

### 2.6. Analysis of Mass-Spectrometric Data

We merged the result of MS analysis from different bands to one large dataset and used the summarized detected peptide spectrum matches (PSM) that reflect the abundance of proteins in one lane. We used a threshold of ≥5 for PSM, i.e., omitted hits where PSM were below 5 in either non-stressed or stressed samples. In our further analysis, the observed PSM values were used for comparing the amount of cross-linked proteins before and after stress. For normalizing the amount of cross-linked proteins, we used the *E. coli* protein abundance dataset of PaxDb [38]. The whole working dataset of the MS data complemented by protein abundance is listed in Table S3 (Supplementary Materials).

Proteins were classified into two separate groups: (1) proteins of increased interaction potential (IIP), for which the observed PSM value increased at least two times upon stress, and (2) proteins of decreased interaction potential (DIP), for which the observed PSM value after stress was at the maximum half of that of its pre-stress value. We calculated the total number of cross-linked proteins before and after heat stress using raw and normalized data as well (Table S3 (Supplementary Materials)).

### 2.7. CD Spectroscopy and Thermal Denaturation Experiments

Circular dichroism (CD) spectroscopy experiments were carried out on a Jasco J-810 instrument (Japan Spectroscopic Co., Tokyo, Japan) equipped with a Peltier-controlled thermostat. For secondary structure determination in 10 mM Na-phosphate, pH 7.4 buffer and in the presence of 30% trifluoro-ethanol (TFE), a quartz cell of 10 μM pathlength was used with a protein concentration of 5 mg/mL. Four scans were accumulated by using a scanning rate of 20 nm/min, a bandwidth of 1 nm, and a data integration time of 4 s. Secondary structure composition was analyzed by the BeStSel algorithm [39].

For thermal denaturation studies of 2 μM citrate synthase in the presence of various concentrations of WT and Full-Scr ERD14, a 1 mm pathlength cell was used. Thermal scans were carried out in the temperature range of 10–80 °C with a scanning rate of 2 °C/min. Thermally induced transitions were monitored by the ellipticity at 220 nm, which is characteristic for the α-helix structural component of citrate synthase. Experiments were carried out at least in duplicates. A two-state transition model described by [40] (Prot. Sci. 1995) showed a good fit to the denaturation curves and provided the melting temperature (*T_m_*) and enthalpy change of unfolding (Δ*H*_m_). We have to note that heating to 80 °C made the unfolding of citrate synthase irreversible, indicating that the results are rather limited to comparison of the samples than interpreting the absolute values.

### 2.8. Statistical Analysis

To assess the statistical significance of the observed differences, a large number of intra- and inter-experiment replicates were done. The survival rates showed minimal standard deviation within the same experiment, and only slightly larger deviation between experiments with a Gaussian distribution. Unpaired *t*-tests were used to quantify the significance of the differences between survival rates of the differently transformed bacteria.

Statistical analysis of the MS results was performed in R programming language using RStudio graphical interface.

## 3. Results

### 3.1. ERD14 Protects Cells under Temperature Stress

In vitro observations of the chaperone activity of IDSPs, such as ERD14 [10], do not ascertain their chaperone activity in a cellular context. To be able to address the chaperone activity of ERD14 on a whole-cell level, we set up an in vivo assay based on measuring the viability of *E. coli* cells by following cell proliferation before and after applying a transient heat stress (Figure 1A). This stress (50 °C × 15 min) causes a significant reduction of the number of viable cells, in which overexpression of the globular control protein GST (survival rate 38.9%) or the IDP calpastatin [10] (survival rate 38.7%) offers no help (Figure 1B). On the contrary, if ERD14 is overexpressed, the survival rate of *E. coli* cells following stress remains 73.9% (all survival rate data are enlisted in Table S5 (Supplementary Materials)).

Previous analyses suggested two possible molecular mechanisms for the chaperone activity of IDPs: (1) one that assumes the physical separation of misfolded substrate molecules by disordered chaperone chains without (specific) physical contacts (a basic molecular shield mechanism [22]) and (2) another one that relies on transient contacts between the proteins (entropy transfer model [9] or an extended molecular shield model [7]). To have a first glimpse at the possible explanation of the protective effect of ERD14, we asked how abolishing local recognition elements while preserving the overall amino acid composition and the disordered state (Figure S3 (Supplementary Materials)) affects its function. To achieve this, we created a variant of ERD14 by scrambling the entire sequence of the protein (for all ERD14 constructs, see Supplementary Information and Table S1 (Supplementary Materials)). This protein, Full-Scr ERD14, offers no protection to cells against heat stress (survival rate 39.8%, Figure 1B). This, and the lack of protection by the fully disordered calpastatin, points to the interplay of partner binding and structural disorder in the in vivo chaperone effect of ERD14. As suggested by the literature, this cellular effect could originate from protecting membranes and/or other cellular proteins [41,42].

### 3.2. ERD14 Is Largely Disordered In Vivo

Several LEA proteins and most hydrophilins have been shown to be structurally disordered proteins [7,9,16,19]. Although their chaperone effect has been amply demonstrated in vitro and also in vivo, the direct functional role of their structural disorder has never been addressed. In our prior in vitro multidimensional NMR analysis, we could assign 181/185 residues of ERD14 (cf. BMRB entry 16876) and showed that the protein is fully disordered, with some tendency of its conserved motifs to sample transient secondary structures [31]. Of course, due to extreme molecular crowding in the cell, which is primarily caused by very high protein concentrations [43], the structural features of ERD14 in vitro do not necessarily translate to its behavior in vivo, although for several in-cell IDPs, NMR suggested a primarily disordered in vivo state [44,45,46]. However, it is rarely asked how the disordered state observed in the cell actually pertains to the function of the protein [35].

**Figure 2:**
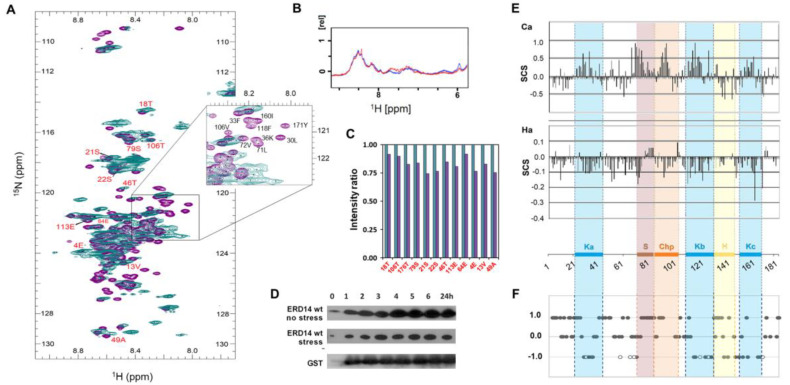
ERD14 is largely disordered in the cytoplasm by in-cell NMR. (**A**) ERD14 was overexpressed in *E. coli* cells under ^13^C- and ^15^N-labeling conditions and its in-cell ^1^H-^15^N HSQC spectrum (teal) was recorded at 700 MHz, T = 277 K. The spectrum is overlaid onto the reference HSQC spectrum (purple) recorded of purified ERD14 at 700 MHz, 277 K at pH 7.7 (cf. Figure S2C (Supplementary Materials)). The majority of the peaks are in good accordance, with certain peaks missing/disappearing due to extensive line broadening (assignment for some peaks is shown, for a complete list, cf. Table S1 (Supplementary Materials)). The narrow region in which peaks are distributed in the proton dimension (8.1–8.8 ppm) indicates that the protein is largely disordered in the cytoplasm of the cell. The region most affected by cellular conditions and interaction partners is also shown as a blow-up. (**B**) Wild-type (WT) ERD14 is stable in cells during the NMR measurements: overlaid 1D spectra (first day (blue) and 5th day (red)) show no significant change in signal intensity. Cell viability remains after the in-cell NMR measurements: after inoculation of the sample into fresh media, the cells are capable of growing. (**C**) Comparison of NMR signal intensities within the cell (teal) and after extraction and purification (purple). Twelve peaks (labeled red on 2A) well-defined both in vivo and in vitro were selected for analysis. (**D**) ERD14 is stable under the conditions of heat stress in the cytoplasm of *E. coli* cells, as shown by Western blot of FLAG-tagged ERD14 induced by IPTG (at t = 0) following the termination of its expression by the removal of IPTG (at t = 30 min) with and without stress. In comparison, Western blot of GST control is also shown. (**E**) Previous in vitro NMR studies show that conserved regions of ERD14 sample transient helical structures in isolation: sequence corrected Secondary Chemical Shift (SCS) values. Areas where the Cα values are shifted upfield, while the Hα values are shifted downfield, correspond to partially formed helices (figure partly adapted from [31]). Conserved motifs of EDR14 are marked by colored shading. (**F**) Based on the assignment and analysis of the in-cell spectrum, we mapped a comparison of in-cell and in vitro NMR spectra on the ERD14 sequence (cf. Suppl. Table S2 (Supplementary Materials)). “Steady” (+1) peaks do not change position, “disappearing” (−1) peaks are not seen in vivo, empty circles represent residues that are disappearing in the presence of high dextran concentration, whereas “broadening” (0) peaks broaden and overlap so much that their identity is difficult to determine in vivo.

Therefore, to address if ERD14 is disordered in cells and to find out if its potent cellular chaperone activity is linked with its structural disorder, we overexpressed ERD14 in *E. coli* cells under isotope labeling conditions (in M9 minimal medium, supplemented by ^15^NH_4_Cl, Materials and Methods) and recorded its in-cell ^1^H-^15^N-HSQC spectrum at 277 K (Figure 2A). Although spectral details are affected by cellular crowding and the inherent inhomogeneity of the cell suspension (causing broadening and disappearance of signals), the in vivo spectrum overlaid onto the corresponding in vitro spectrum shows that the protein is largely disordered in the cell. The narrow distribution of peaks observed in the proton dimension (8.1 ≤ δ ≤ 8.8 ppm) represents a distinctive feature of IDPs both in vitro and in vivo [47]. To show that ERD14 does not leak from cells during the in-cell NMR experiment [48,49], we have taken the NMR sample once the in-cell spectrum was recorded, centrifuged it (13,000× *g* for 2 min), and recorded the HSQC spectrum of its supernatant (Figure S4A (Supplementary Materials)). An SDS-PAGE gel was also run to directly visualize the protein content of cells and supernatant (Figure S4C (Supplementary Materials)). Both the gel picture and NMR spectrum show the absence of labeled protein in the supernatant of cells, confirming that overexpressed ERD14 stays inside cells for the duration of the entire experiment and is indeed disordered in the crowded cytoplasm.

The region covered by the HSQC spectrum suggests that the protein is in an overall disordered state, and its certain regions do not bind to partners, whereas its other regions are much affected by binding-induced line broadening, causing the disappearance of peaks (Figure 2A, zoomed region). To prove that we see 100% of the molecules in the cell [50,51,52,53], i.e., disordered ERD14 conveys cellular function, we have quantified and compared peak intensities in the cell and following extraction (Figure 2C). The comparison shows that the intensity of select peaks (clearly identifiable both in the cell and after extraction) decrease only slightly upon purification; i.e., we have the NMR signature of the functional protein molecules in the cell.

Therefore, these experiments connect the disordered state of an IDP with its in vivo function, which goes beyond interpreting IDP function by induced folding when (part of) the protein becomes structured in the presence of a partner [32].

### 3.3. ERD14 Is Stable in the Cell under Normal and Stress Conditions

As a caveat to this link between structural disorder and the protective (chaperone) effect of ERD14 in live cells, one has to assume that the protein is stable in the cell under stress, which might be in conflict with the noted sensitivity of IDPs to proteolytic degradation [54]. To this end, we recorded the 1D ^1^H spectrum of ERD14 in cells on the first and fifth day after expression (Figure 2B). The apparent lack of observable changes suggests no degradation of the protein. This is also corroborated by arresting protein translation and applying stress conditions under which the protein is an effective chaperone, followed by Western blot with an anti-ERD14 antibody (Figure 2D). Apparently, ERD14 is about as stable as the globular control protein glutathione S-transferase (GST), without a discernible decay for up to about 24 h, even after heat stress. It is to be noted that similar stability was also reported for a few other IDPs in in-cell NMR experiments [44,45,46].

### 3.4. Conserved Regions of ERD14 Bind to Partner Molecules in the Cell

In previous in vitro NMR experiments [30], secondary chemical shift and relaxation data showed that the conformation of ERD14 is close to a random coil, with its five short conserved segments (Ka, Kb, Kc, S, and ChP) transiently sampling local helical conformations (Figure 2E). Given the suggested in vitro functions of similar K segments in other dehydrins [55,56,57] and the general tendency of structurally pre-formed regions (i.e., pre-structured motifs, PreSMos [33]) in IDPs to be involved in molecular function, we hypothesized that these five conserved [6,8] regions may be involved in the functional interactions of ERD14.

Therefore, we have set out to analyze the in vivo HSQC spectrum of ERD14 in detail. To this end, first, we transferred the assignment from our prior in vitro NMR analysis to conditions similar to those within *E. coli* cells, where macromolecular crowding and relatively high pH conditions (about pH 7.6–7.8 [58]) prevail. Through a series of spectra recorded at various temperatures (277 K to 288 K, Figure S1A (Supplementary Materials)) and pHs (pH 6.58 to 7.70, Figure S1B (Supplementary Materials)), the assignment of resonances could be transferred to our in vitro reference HSQC spectrum (T = 277 K, pH 7.7: Figure S3C (Supplementary Materials), BMRB Accession Number 26636).

A comparison of this in vitro spectrum and the in vivo ^1^H-^15^N-HSQC spectrum of ERD14 (Figure 2A) shows that several characteristic peaks occupy the same position in vitro and in vivo (42 resonances, marked as “steady” in Table S2 (Supplementary Materials)). Thus, these residues must be in a comparable (disordered) structural state in vitro and in vivo. Second, a subset of the signals vanishes from the in-cell spectrum (29 “disappearing” resonances), which is probably due to severe line broadening caused by partner binding, because only 5 of them disappear under crowding conditions in vitro elicited by 400 mg/mL dextran (cf. Figure S2 (Supplementary Materials)). Third, due to the high degree of signal overlap of the central spectral region, many resonances cannot be resolved with high confidence, but they appear to be largely preserved (95, marked as “broadening” in Table S2 (Supplementary Materials)). Finally, 19 resonances cannot be linked to the in vivo spectrum; thus, they are marked as “unknown” and are left out of further consideration. We interpret the disappearance of resonances as a result of the binding of ERD14 segments to other macromolecular partners, i.e., protein molecules or membrane structures in the cell, whereas we relate their steady or broadening behavior as a sign of a primarily disordered state in the cell (Figure 2F).

**Figure 3:**
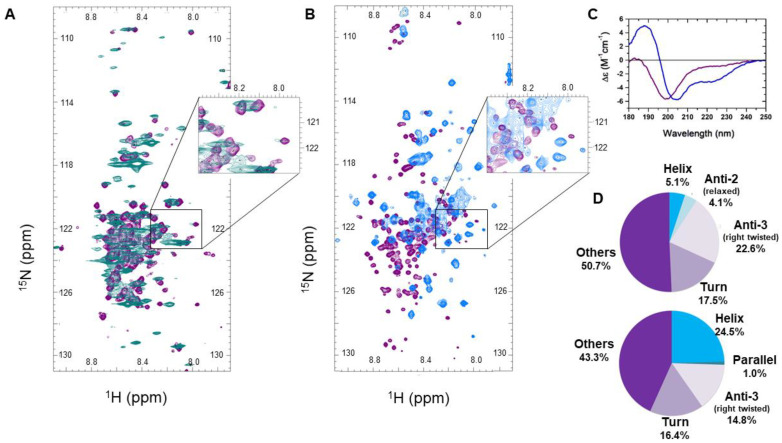
Changes in the in-cell NMR spectrum of ERD14 correspond to intracellular interactions (**A**) In-cell ^1^H-^15^N HSQC spectrum of scrambled (Full-Scr) ERD14 (teal) is compared to the HSQC spectrum (purple) of purified Full-Scr ERD14 recorded in buffer. Broadenings of several resonances can be observed, but there is no peak disappearance in the region observed for WT ERD14 (insert, cf. also Figure 2A). For easier comparison, the zoomed region is the same as in case of WT ERD14; however, because of the scrambling, the exact sequences are not the same in the two proteins. An assignment of Full-Scr can be found in Supplementary Figure S1C (Supplementary Materials). (**B**) Comparison of the ^1^H-^15^N HSQC spectra of WT ERD14 measured in MES buffer (pH 6.5) (purple) and in the presence of 30% trifluoro-ethanol (TFE) (blue). As shown by circular dichroism (**C**,**D**) measurements (**C**), TFE at this concentration induces conformational changes in ERD14, yet it only causes peak shifts and broadening, no signal disappearances (Panel (**B**), insert). (**D**) Analysis of circular dichroism (CD) spectra by BeStSel [59] suggests that WT ERD14 is mostly disordered (a-helix content 5%), whereas in the presence of 30% TFE, it assumes a significant secondary structure (α-helix content is 24%).

However, such spectral effects can also have other explanations; i.e., they can be accounted for by kinetic-exchange phenomena between different conformational states in the viscous and crowded cytoplasm. We have three lines of evidence against this interpretation. First, we refer to the comparison of the in-cell spectrum of ERD14 (Figure 2A) with that of Full-Scr ERD14 (Figure 3A), which has no cellular protective effect (Figure 1B) and lacks the preformed motifs. Whereas the overall disorder of the two proteins is very similar (IUPred [59] predictions on Figure S3 (Supplementary Materials)), the peak disappearance caused by line broadening is not observable in the Full-Scr ERD14 spectrum (Figure 3A). We could add that similar observations were made for other IDPs expressed in heterologous systems, e.g., alpha-Synuclein [44,48] and tau protein [34], which also argues against cellular crowding causing the disappearance of HSQC peaks. Second, we recorded the HSQC spectrum of purified ERD14 under increasingly viscous, crowding conditions by adding dextran to the solution at varying concentrations from 0 to 400 mg/mL in vitro [60,61] (Figure S2 (Supplementary Materials)). The dextran applied (Mw = 70 kDa) causes a crowding effect, which is often used to mimic intracellular conditions [60,61,62] and usually has similar effects to other crowders, such as Polyethylene glycol (PEG), Ficoll, or globular proteins [44,63]. However, under these conditions, only a few resonances get shifted and/or broadened (8, marked on Figure S2 (Supplementary Materials)), and only 5 of them disappear (marked on Figure 2F by empty circles). Third, we also applied 30% trifluoro-ethanol (TFE), which is an agent that promotes the secondary structure formation of (disordered) proteins [64] and was noted to stabilize transient α-helices in dehydrins [65]. Whereas peaks in the region of the HSQC spectrum of ERD14 most affected by cellular conditions (Figure 2A) only broaden and shift but do not disappear in the presence of 30% TFE (Figure 3B, spectra of TFE titration shown on Figure S5 (Supplementary Materials)), circular dichroism (CD) shows that the helical content of ERD14 increases significantly (from about 5% to 25%, Figure 3C,D. In a comparable system, the alpha-helix content of the transactivator domain of p53 is induced by cellular crowding [35], but it also does not cause the disappearance of peaks from its in vivo spectrum. In all, we may conclude that a severe shift or disappearance of peaks in the in-cell spectrum (Figure 2A and Table S2 (Supplementary Materials)) can be attributed to the binding of ERD14 to high-molecular weight partner molecules (proteins, membrane components or nucleic acids) in the cell (as further confirmed by pulldown-MS experiments; see later).

To identify the regions involved, the differences between in-cell and reference spectra were projected onto the sequence of ERD14 (Table S2 (Supplementary Materials)) and visualized by plotting +1 for “steady”, 0 for “broadening”, and −1 for “disappearing” residues (Figure 2F). By also considering resonances that disappear in the presence of 400 mg/mL dextran, we found that the binding of partners can be associated with the K-segments (Ka, Kb, and Kc) and partially the Chp and H segments. Segment S seems to remain disordered; i.e., it is not involved in partner binding, and the same applies to some flanking regions, primarily to the N- and C-termini of the protein, and the long linker between Ka and S segments (Figure 2F). This pattern underscores the possible functional involvement of these regions in the protection function of ERD14.

### 3.5. Improved Cell Viability Is Due to Protein Protection

**Figure 4:**
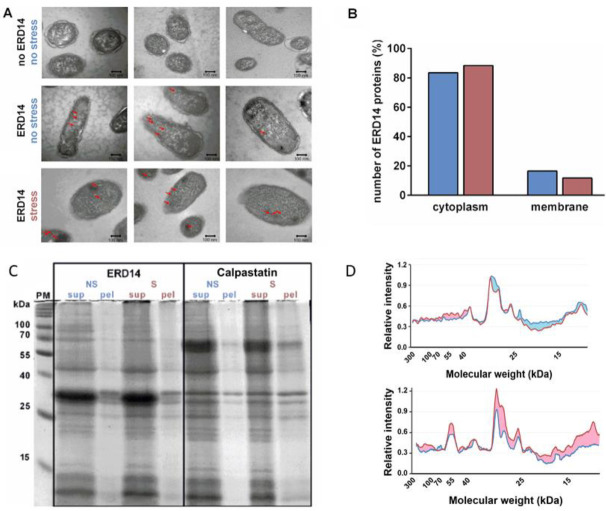
ERD14 protects the proteome of the cells. (**A**) ERD14 is primarily localized in the cytoplasm and does not translocate to the cell membrane upon stress, as shown by electron microscopy (EM). FLAG-tagged ERD14 was overexpressed in *E. coli* cells, which were fixed by formaldehyde either before (second line) or after (third line) heat stress (50 °C × 15 min). Slices of fixed cells were immunostained by anti-FLAG primary and nanogold-conjugated secondary antibody. Red arrows show gold particles corresponding to ERD14 protein, indicating a primarily cytoplasmic localization both before and after stress. No staining is seen in cells in which ERD14 was not overexpressed (first line). (**B**) Analysis of 100 gold particles show that 84% and 88% of the observed particles are localized in the cytoplasm before (blue) and after (purple) heat stress, respectively, whereas the low percentage of particles localized next to the membrane does not increase upon stress. (**C**) ERD14 protein has a general protective effect on the proteome, as shown by the pattern of proteins in solution (supernatant, sup) and pellet (pel) on 12.5% SDS-PAGE gels in cell extracts of cells overexpressing ERD14 or calpastatin, before (no stress, NS) and after (stress, S) heat stress. (**D**) Densitometric analysis of pellets before (blue line) and after (red line) stress was carried out using ImageJ image processing software. Differences are colored for better clarity. The results suggest that after heat stress, proteins within the whole Mw range get more aggregated in the presence of calpastatin but remain more in solution in the presence of ERD14 (the observed differences are significant).

Our in-cell NMR results clearly show that certain regions of ERD14 engage in partner binding. To understand the cellular mechanism of the protective effect of dehydrins, we need to know the nature of these partners. Although in previous in vitro studies, we observed no effect of ERD14 on membrane fluidity [7,10], and the cellular protection function of dehydrins is mostly attributed to the protection of protein partners [7,10,20,21,22], we asked if preferential membrane-proximal localization could point to the protection of membranes by ERD14. To this end, we expressed FLAG-tagged ERD14 in the cells and performed immunogold labeling after fixation and embedding (Materials and Methods). Transmission electron microscopy (TEM) images of immunoreactive ERD14 molecules (Figure 4A) suggest a cytoplasmic localization of the disordered chaperone prior to stress, which does not change upon applying the heat stress (50 °C × 15 min) to cells (Figure 4A,B). In conjunction with our prior observations [7,10,20], this result suggests that membrane stabilization is not the preferred mechanism by which ERD14 protects cells against the heat stress applied. This points to proteins being the primary clients of this dehydrin, which is in line with a range of prior studies [7,10,20,21,22]. Therefore, we next asked if, after stress, a larger fraction of the proteome gets aggregated in cells overexpressing the non-protective control protein calpastatin (cf. Figure 1B) than in cells overexpressing ERD14. To this end, we treated cells expressing either ERD14 or calpastatin with heat stress, and run their proteins in supernatant and pellet, separated by centrifugation, on SDS gel electrophoresis (Figure 4C). Quantitative analysis by densitometry (Figure 4D) shows a proteome-wide protection from aggregation by ERD14.

These results suggest that the protection of cells by overexpressed ERD14 is due to protecting either the entire proteome or a very wide range of specific proteins.

### 3.6. ERD14 Has Multiple Protein Partners in the Cell

**Figure 5:**
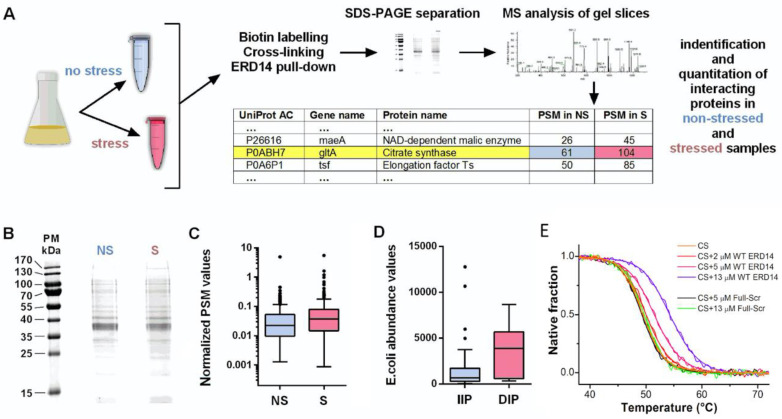
Identification of potential interaction partners of ERD14. (**A**) Brief overview of the identification of the binding partners of ERD14 in the cell: biotinylated ERD14 was overexpressed and cells were treated with a membrane-permeable cross-linker, either without or with prior exposure to heat stress (50 °C × 15 min). Protein partners bound to ERD14 were affinity purified with Streptavidin–Sepharose, separated on SDS-PAGE, in-gel digested by trypsin, and analyzed by LC-MS/MS (for details, cf. Materials and Methods, for data, cf. Table S3 (Supplementary Materials)). (**B**) SDS-PAGE of cross-linked proteins without (NS) and with (S) stress treatment. (**C**) The distribution of normalized copy numbers of proteins (peptide spectrum matches, or PSM) cross-linked to ERD14 before and after heat stress shows an increased number of proteins interacting with ERD14 after stress (*p* = 0.004). (**D**) Average cellular abundances of the identified proteins with increased (IIP) or decreased (DIP) ERD14 interaction potential are significantly different (*p* = 0.0002), showing that increased binding to ERD14 upon stress is not due to protein abundances. (**E**) Thermal denaturation of citrate synthase in the presence of ERD14 reveals their interaction. First, 2 μM citrate synthase (CS) was heated up in the presence of various concentrations of WT ERD14 (0—orange, 2—red, 5—magenta and 13 μM—purple) or its scrambled variant (5—black and 13 μM—green). Thermal denaturation was followed by the CD signal at 220 nm. The presence of WT ERD14 increased the melting point of CS and altered the sharpness of the unfolding transition in a concentration-dependent manner. Despite its similar physicochemical properties, the Full-Scr ERD14 variant had no relevant effect on the thermal denaturation of CS. Normalized thermograms are shown. Data was fitted according to Shih et al. [40].

The above results can be best interpreted by assuming that ERD14 protects many non-specifically binding proteins, the effect of which appears at the level of the entire cell. The most direct approach to support this interpretation is to identify binding partners of ERD14 under stress conditions. To this end, we have carried out a proteomic analysis by overexpressing biotin-labeled ERD14 in cells, stabilizing its transient protein–protein interactions with dithiobis(succinimidyl propionate) (DSP, Lomant’s Reagent) cross-linker [66] in both stressed and non-stressed cells, and identifying cross-linked proteins from SDS-PAGE gel by mass spectrometry (MS, see scheme in Figure 5A).The cross-linking reaction was quenched after 7 min, to suppress non-specific cross-linked products, and cross-linking was reverted prior to SDS gel electrophoresis (Figure 5B). MS protein identification was performed on the total lane by label-free comparison of protein abundance in stressed and non-stressed samples, which showed that the total number of peptide hits (peptide spectral matches, PSMs) before (*n* = 6965) and after (*n* = 11,138) stress are significantly different (Welch Two-Sample *t*-test on logarithmic data, *p*-value = 0.004, Figure 5D). As also observed with traditional and well-studied chaperones (e.g., Hsp90 and GroEL/GroEs [67,68]), these results suggest that the chaperone is in contact with its clients even under normal conditions, but there is a significant increase in ERD14-bound proteins when cells encounter stress (Figure 5C).

The MS analysis of cross-linked proteins brought up 255 hits (Table S3 (Supplementary Materials)), 76 of which show significantly increased (increased intensity protein, IIP), while 15 show significantly decreased (decreased intensity protein, DIP) interaction potential with ERD14 upon stress (the top 20 IIP and 10 DIP proteins are also presented in Table S4 (Supplementary Materials)). By looking for the natural abundance of ERD14 interactors in the PaxDb database [38], we found that the proteins in the IIP group have significantly lower *E. coli* abundance than the ones in the DIP group (Welch Two-Sample *t*-test on logarithmic data, *p*-value = 0.0002, Figure 5D). Binding more low-abundance proteins and less high-abundance proteins after stress suggests that ERD14 binds to proteins—more specifically, probably to proteins with structural defects to keep them soluble. The top 20 IIP proteins have significantly lower isoelectric points (mean pI: 5.66) than the top 10 DIP proteins (mean pI: 8.96) (Student’s independent sample *t*-test, *p* < 0.0001), indicating that acidic proteins tend to bind stronger to ERD14 during heat stress. This observation underlines the importance of the lysine residues of the K-segments in protein binding.

### 3.7. In Vitro Protection of an In Vivo Partner

The above cross-linking and pull-down studies show that ERD14 binds many protein partners in the cell (Table S3 (Supplementary Materials)). Out of 76 stress-dependent binders (IIP), we selected citrate synthase (CS) to demonstrate in vitro that ERD14 binding does protect it against heat stress. CS is frequently used in in vitro chaperone assays, showing that different LEA proteins (e.g., AavLEA and RcLEA) can preserve its enzyme activity and/or prevent its aggregation under stress elicited by drying, freezing, or high temperatures [7,21]. Therefore, to determine whether CS is stabilized by ERD14 in vitro under heat stress, thermal denaturation of CS was measured by circular dichroism (CD) spectroscopy at 220 nm in the presence of various concentrations of WT ERD14 or the Full-Scr ERD14 variant (Figure 5E). CS exhibited a sigmoid-like, cooperative unfolding transition around 50 °C. In the presence of WT ERD14, the melting temperature was increased from 49.5 °C (CS alone) in a concentration-dependent manner to 55.3 °C (13 μM WT ERD14, Table 1). Assuming a two-state transition, the enthalpy change of unfolding (Δ*H*_m_) was calculated by fitting to the raw data as described by Shih et al. [40]. Although the unfolding of CS was not reversible upon heating to 80 °C, the two-state model provided a perfect fit, and we used the results for comparative purposes.

In the function of the WT ERD14 concentration, Δ*H*_m_ is decreased from 475 kJ/mol (CS alone) to 316 kJ/mol (13 μM WT ERD14, Table 1). Δ*H*_m_ is related to the disruption of the stabilizing interactions upon unfolding. A decrease in Δ*H*_m_ in the presence of WT ERD14 might indicate that upon unfolding, CS makes interactions with ERD14, providing a contribution to Δ*H*_m_ with the opposite sign. To investigate if the observed effects represent interactions with ERD14 in a sequence-specific manner and are not a result of changes in the environment or crowding effect, we studied the influence of the Full-Scr ERD14 variant on the thermal denaturation of CS. In the presence of up to 13 μM Full-Scr ERD14, CS exhibited no significant differences in its thermal denaturation profile, indicating that the observed effects of WT ERD14 on CS cannot be derived from the environmental changes and general physicochemical properties of the hydrophilic ERD14 chain, but rather, sequence-specific interactions occur.

## 4. Discussion

Whereas complete understanding of the function of a plant chaperone requires studies in plant cells, the detailed structural studies conducted here require the use of an *E. coli*-based system, since the application of bacterial cells enables us to carry out structural studies by in-cell NMR, which is not yet possible in plant cells [36,69]. Studying a stress-related IDP enables us to overcome also the problem caused by the low sensitivity of NMR and low physiological concentrations of disordered signaling proteins [44,48], because the high intracellular concentrations required for in-cell NMR are in the physiological range of IDSPs attained under stress conditions [6,30].

The link of its molecular activity with residual disorder inside cells has many intriguing consequences for the cellular mechanism(s) of IDPs in general and IDSPs in particular. First, we show that ERD14 is largely disordered in vivo, which suggests that IDPs might not be made to fold by crowding in the cell; i.e., structural disorder is their native, physiological state. This novel insight underscores that structural disorder can be compatible with protein function in a cellular context [47], which has important implications.

### 4.1. Disordered Functional State Observed by In-Cell NMR

The overall similarity of in vitro and in vivo spectra of ERD14 shows that ERD14 is mostly disordered in the cytoplasm of a living cell that it protects against stress, which substantiates that intrinsic disorder is the native, functional state of this chaperone. Further, in-cell NMR shows that the protein is not completely disordered, but it contains dispersed motifs connected by flexible linkers (such as beads on a string), which may be a general function-related organizational principle of many IDPs in the cell [70]. This result adds to a series of studies on the structural state of IDPs under the extreme macromolecular concentrations of the interior of the cell [34,35,37], which was thought to make IDPs fold [3] and shows that the native state of IDPs may in fact be disordered. The novelty of our results stem from connecting the demonstrated structural disorder with the function of this protein.

### 4.2. Structural Disorder and Stability of IDPs In Vivo

The in vivo stability of IDPs is yet another critical issue in the literature: their open and exposed structure makes them very sensitive to degradation in vitro and may predispose them for rapid degradation in vivo [54]. The fact that the in vivo half-life of proteins does not strongly correlate with structural disorder [71] has led to suggestions that the level of IDPs is tightly regulated at many levels [72], and they might be protected by binding partners [32]. In addition, in a few cases, in-cell NMR data provided direct evidence not only for their structural disorder but also their stability in the cell in lengthy NMR experiments [34,35,36]. Our new results also strongly argue—at least for ERD14—that its cellular disorder does not result in rapid degradation, which is probably due to specific sequence features and the general lack of uncontrolled proteolytic activity in the cell [73].

### 4.3. Membrane Versus Protein Protection of ERD14

LEA proteins and other hydrophilins are effective chaperone-like proteins under a variety of stress conditions both in vitro and in vivo and emerge as a new class of chaperones [8,41,42,74]. In a few, mostly in vitro, studies, they have also been suggested to bind and maybe protect membranes [75], but the overwhelming majority of results point to their effect on proteins [7,20]. Our results here are also consistent with the protein chaperone activity of ERD14. EM data do not concur with the membrane localization of ERD14 either before or after stress, whereas protein binding and protection studies suggest the protection of a broad range of proteins, as illustrated by the proteome protection in Figure 4 and Figure 5. In all, our results add solid evidence to the expanding view that IDSPs represent a novel class of protein chaperones [8,41,42,74].

Having observed this effect on bacterial proteins by a plant dehydrin raises the issue of interaction specificity; however, this is often encountered in the chaperone field. Actually, we should not infer classical protein–protein interaction specificity here, as chaperones in general are known to bind a broad range of clients (hundreds, as shown for e.g., Hsp90 and GroEL/GroEs [67,68]). In accord, the chaperone field presents many experimental results of heterologous systems, both in vitro with biologically unrelated clients (e.g., firefly luciferase), or in vivo in heterologous cellular systems (e.g., yeast). This has been also the case with plant dehydrins, having been shown to protect bacterial, yeast, or even mammalian cells [20,21,22,23,24,25,27].

Our pull-down experiments corroborate the assumption that ERD14 is able to bind several protein partners both before and during stress. Since IDPs are well known to be able to bind partner molecules in a fast and transient manner [76], this binding does not necessarily interfere with the normal function of proteins as shown by the fact that the activity of neither CS [77] nor catalase [78] is reduced in the presence of ERD14 (Figure S6 (Supplementary Materials)) and that the growth rate of ERD14-expressing *E. coli* cells is similar to the controls (Figure S7 (Supplementary Materials)). The importance of the sequentially determined protein-binding capacity of ERD14 was further confirmed by the effect of ERD14 on the thermal unfolding of CS (Figure 5E). The inability of the Full-Scr ERD14 variant to modify the melting temperature of CS argues against the general osmolyte effect of the protein. Since Full-Scr ERD14 has an amino acid composition identical to WT ERD14, only with a randomized sequence, it should provide the same protection, were it dependent on the osmolyte and crowding effect.

Since CS is not an essential protein for *E. coli*, its protection would not in itself explain a commensurable protection of the cell under stress; rather, it argues for a binding-related, broad effect of ERD14 on non-specific partners.

### 4.4. The Molecular Mechanism of a Disordered Chaperone In Vivo

**Figure 6:**
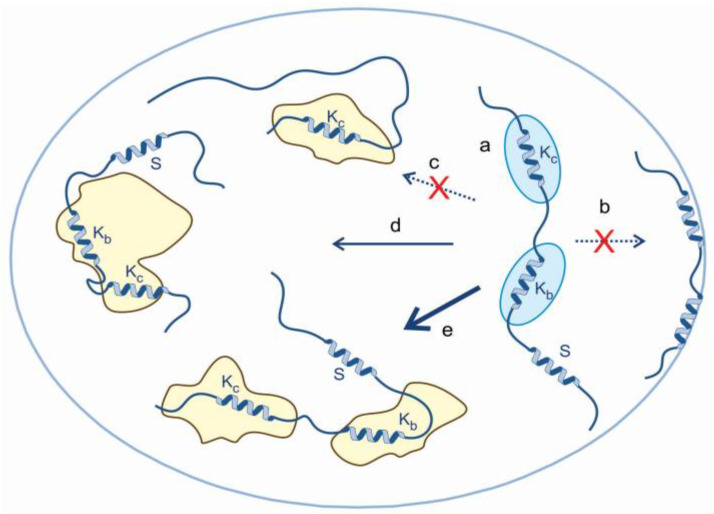
A general mechanistic model of the chaperone activity of ERD14 in vivo. Our in vivo observations alongside previous in vitro data on the protection of ERD14 of the proteome can be rationalized by a model that combines the binding functions of short conserved motifs and space filling (entropic separation) by disordered linking and flanking regions. The model incorporates the following elements: (**a**) under non-stressed conditions, largely disordered ERD14 is loosely associated with native proteins, with its multivalent binding is mediated by its short binding regions (exemplified by Kb and Kc), while its S segment is unbound. Upon stress, many proteins in the cell become structurally compromised and expose hydrophobic regions; thus, ERD14 can engage with them, rather than the cell membrane (**b**). The binding of only one conserved segment is not compatible with our NMR results (**c**), whereas multivalent binding protects protein partners and the cell. Binding to one partner cannot be ruled out (**d**), but effective protection is probably achieved by simultaneously binding more than one partner (**e**).

To integrate our observations, we suggest a mechanistic model of the in vivo chaperone action of ERD14 (Figure 6). A large part of the protein is disordered, while its transiently folded segments engage in partner binding. ERD14 might be in loose association with proteins prior to stress (Figure 6a), which is also evident for traditional chaperones [67,68]. This pre-association shows that chaperones are continuously at work even under non-stressed conditions, which is significantly boosted by deterioration in the state of the proteome elicited by stress. This mechanism is not compatible with models in which the chaperone does not have any physical contact with its clients, such as a space filler or macromolecular osmolyte. The association of ERD14 is multivalent (either to the same (Figure 6d) or multiple partners (Figure 6e), as follows from the disappearance of its multiple motifs from the in-cell NMR spectrum. By such a binding mode as outlined, ERD14 can hold different proteins apart, but parts of the same protein in spatial proximity, in a (re)folding competent form. It can also solvate its partners and prevent the exposure of their hydrophobic regions amenable for aggregation. As a disordered protein, ERD14 cannot consume ATP energy; it cannot function as an active “foldase” [79], but it can hold its structurally compromised client at bay, until spontaneous re-folding succeeds. Such “holdase” activity is consistent with the extended molecular shield [7] and entropy transfer [9] models of chaperone action of IDPs. The protection of a large part of the proteome and the capability of the protein to protect bacterial cells against heat stress underlines that the effect of ERD14 as a chaperone is of broad specificity. Again, this is not unusual in the chaperone field, as even traditional chaperones have hundreds of clients [67,68].

## Figures and Tables

**Table 1:** Melting points (*T_m_*) and unfolding enthalpies (Δ*H*_m_) obtained by fitting to the raw data following the methodology of Shih et al. [40].

	CS	CS + 2 μM WT ERD14	CS + 5 μM WT ERD14	CS + 13 μM WT ERD14	CS + 5 μM Full-Scr	CS + 13 μM Full-Scr
*T_m_* (°C) ± SE	49.5 ± 0.1	50.5 ± 0.4	51.9 ± 0.1	55.3 ± 0.3	49.5 ± 0.2	49.9 ± 0.1
Δ*H* (kJ/mol) ± SE	475 ± 24	396 ± 7	349 ± 11	316 ± 18	474 ± 19	460 ± 4

## Supplementary Materials

The following are available online at https://www.mdpi.com/2073-4409/9/8/1856/s1, Supplementary Methods, Figure S1. HSQC spectrum of WT ERD14 at different temperatures and pHs, Figure S2. HSQC spectrum of ERD14 under crowding conditions, Figure S3. Disorder prediction of WT ERD14 and Full-Scr ERD14, Figure S4. ERD14 does not leak from *E. coli* cells during in-cell NMR experiment, Figure S5. HSQC spectrum of ERD14 in the presence of increasing TFE concentration, Figure S6. In vitro protection of the cellular partners of ERD14, Figure S7. Growth curves of *E. coli* cells expressing different constructs Table S1. Sequences of ERD14 constructs and controls, Table S2. In-cell NMR results, Table S3. Protein partners of ERD14 identified by cross-linking and mass spectrometry, Table S4. Protein partners of ERD14 significantly changing upon heat stress, Table S5. *E. coli* viability measurements.

## References

[1] Tompa P. (2011). Unstructural biology coming of age. Curr. Opin. Struct. Biol..

[2] Van Der Lee R., Lang B., Kruse K., Gsponer J., De Groot N.S., Huynen M.A., Matouschek A., Fuxreiter M., Babu M.M. (2014). Intrinsically disordered segments affect protein half-life in the cell and during evolution. Cell Rep..

[3] Tompa P. (2005). The interplay between structure and function in intrinsically unstructured proteins. FEBS Lett..

[4] Saibil H.R. (2008). Chaperone machines in action. Curr. Opin. Struct. Biol..

[5] Balchin D., Hayer-Hartl M., Hartl F.U. (2016). In vivo aspects of protein folding and quality control. Science.

[6] Artur M.A.S., Zhao T., Ligterink W., Schranz E., Hilhorst H.W.M. (2019). Dissecting the Genomic Diversification of Late Embryogenesis Abundant (LEA) Protein Gene Families in Plants. Genome Biol. Evol..

[7] Chakrabortee S., Tripathi R., Watson M., Schierle G.S.K., Kurniawan D.P., Kaminski C.F., Wise M.J., Tunnacliffe A. (2012). Intrinsically disordered proteins as molecular shields. Mol. BioSyst..

[8] Graether S.P., Boddington K.F. (2014). Disorder and function: A review of the dehydrin protein family. Front Plant. Sci..

[9] Tompa P., Csermely P. (2004). The role of structural disorder in the function of RNA and protein chaperones. FASEB J..

[10] Kovacs D., Kalmar E., Torok Z., Tompa P. (2008). Chaperone Activity of ERD10 and ERD14, Two Disordered Stress-Related Plant Proteins. Plant. Physiol..

[11] Chakrabortee S., Meersman F., Schierle G.S.K., Bertoncini C.W., McGee B., Kaminski C.F., Tunnacliffe A. (2010). Catalytic and chaperone-like functions in an intrinsically disordered protein associated with desiccation tolerance. Proc. Natl. Acad. Sci. USA.

[12] Reichmann D., Xu Y., Cremers C.M., Ilbert M., Mittelman R., Fitzgerald M.C., Jakob U. (2012). Order out of Disorder: Working Cycle of an Intrinsically Unfolded Chaperone. Cell.

[13] Boothby T.C., Tapia H., Brozena A.H., Piszkiewicz S., Smith A.E., Giovannini I., Rebecchi L., Pielak G.J., Koshland D., Goldstein B. (2017). Tardigrades Use Intrinsically Disordered Proteins to Survive Desiccation. Mol. Cell.

[14] Bardwell J.C.A., Jakob U. (2012). Conditional disorder in chaperone action. Trends Biochem. Sci..

[15] Wise M.J., Tunnacliffe A. (2004). POPP the question: What do LEA proteins do?. Trends Plant Sci..

[16] Battaglia M., Olvera-Carrillo Y., Garciarrubio A., Campos F., Covarrubias A.A. (2008). The Enigmatic LEA Proteins and Other Hydrophilins. Plant Physiol..

[17] Reyes J., Rodrigo M.J., Colmenero-Flores J.M., Gil J.-V., Garay-Arroyo A., Campos F., Salamini F., Bartels D., Covarrubias A.A. (2005). Hydrophilins from distant organisms can protect enzymatic activities from water limitation effects in vitro. Plant Cell Environ..

[18] Covarrubias A.A., Cuevas-Velazquez C.L., Romero-Pérez P.S., Rendón-Luna D.F., Chater C.C.C. (2017). Structural disorder in plant proteins: Where plasticity meets sessility. Cell. Mol. Life Sci..

[19] Hundertmark M., Hincha D.K. (2008). LEA (Late Embryogenesis Abundant) proteins and their encoding genes in Arabidopsis thaliana. BMC Genom..

[20] Dang N.X., Popova A.V., Hundertmark M., Hincha D.K. (2014). Functional characterization of selected LEA proteins from Arabidopsis thaliana in yeast and in vitro. Planta.

[21] Zhang X., Lu S., Jiang C., Wang Y., Lv B., Shen J., Ming F. (2014). RcLEA, a late embryogenesis abundant protein gene isolated from Rosa chinensis, confers tolerance to Escherichia coli and Arabidopsis thaliana and stabilizes enzyme activity under diverse stresses. Plant Mol. Biol..

[22] Chakrabortee S., Boschetti C., Walton L.J., Sarkar S., Rubinsztein D.C., Tunnacliffe A. (2007). Hydrophilic protein associated with desiccation tolerance exhibits broad protein stabilization function. Proc. Natl. Acad. Sci. USA.

[23] Wu Y., Liu C., Kuang J., Ge Q., Zhang Y., Wang Z. (2014). Overexpression of SmLEA enhances salt and drought tolerance in Escherichia coli and Salvia miltiorrhiza. Protoplasma.

[24] Ling H., Zeng X., Guo S. (2016). Functional insights into the late embryogenesis abundant (LEA) protein family from Dendrobium officinale (Orchidaceae) using an Escherichia coli system. Sci. Rep..

[25] Swire-Clark G.A., Marcotte J.W.R. (1999). The wheat LEA protein Em functions as an osmoprotective molecule in Saccharomyces cerevisiae. Plant Mol. Biol..

[26] Huang Z., Zhong X.-J., He J., Jin S.-H., Guo H.-D., Yu X.-F., Zhou Y.-J., Li X., Ma M.-D., Chen Q.-B. (2016). Genome-Wide Identification, Characterization, and Stress-Responsive Expression Profiling of Genes Encoding LEA (Late Embryogenesis Abundant) Proteins in Moso Bamboo (Phyllostachys edulis). PLoS ONE.

[27] Li S., Chakraborty N., Borcar A., Menze M.A., Toner M., Hand S.C. (2012). Late embryogenesis abundant proteins protect human hepatoma cells during acute desiccation. Proc. Natl. Acad. Sci. USA.

[28] Reyes J., Campos F., Wei H., Arora R., Yang Y., Karlson D.T., Covarrubias A.A. (2008). Functional dissection of Hydrophilins duringin vitrofreeze protection. Plant Cell Environ..

[29] Arakawa T., Timasheff S. (1985). The stabilization of proteins by osmolytes. Biophys. J..

[30] Kiyosue T., Yamaguchi-Shinozaki K., Shinozaki K. (1994). Characterization of Two cDNAs (ERD10 and ERD14) Corresponding to Genes That Respond Rapidly to Dehydration Stress in Arabidopsis thaliana. Plant Cell Physiol..

[31] Ágoston B.S., Kovacs D., Tompa P., Perczel A. (2011). Full backbone assignment and dynamics of the intrinsically disordered dehydrin ERD14. Biomol. NMR Assign..

[32] Wright P.E., Dyson H.J. (2009). Linking folding and binding. Curr. Opin. Struct. Biol..

[33] Kim D.-H., Han K.-H. (2018). PreSMo Target-Binding Signatures in Intrinsically Disordered Proteins. Mol. Cells.

[34] Bodart J., Wieruszeski J.-M., Amniai L., Leroy A., Landrieu I., Rousseau-Lescuyer A., Vilain J.-P., Lippens G. (2008). NMR observation of Tau in Xenopus oocytes. J. Magn. Reson..

[35] Borcherds W., Theillet F.-X., Katzer A., Finzel A., Mishall K.M., Powell A.T., Wu H., Manieri W., Dieterich C., Selenko P. (2014). Disorder and residual helicity alter p53-Mdm2 binding affinity and signaling in cells. Nat. Methods.

[36] Dedmon M.M., Patel C.N., Young G.B., Pielak G.J. (2002). FlgM gains structure in living cells. Proc. Natl. Acad. Sci. USA.

[37] Theillet F.-X., Binolfi A., Bekei B., Martorana A., Rose H.M., Stuiver M., Verzini S., Lorenz R., Van Rossum M., Goldfarb D. (2016). Structural disorder of monomeric α-synuclein persists in mammalian cells. Nature.

[38] Wang M., Herrmann C.J., Simonovic M., Szklarczyk D., Von Mering C., Wang M. (2015). Version 4.0 of PaxDb: Protein abundance data, integrated across model organisms, tissues, and cell-lines. Proteomics.

[39] Micsonai A., Wien F., Bulyáki É., Kun J., Moussong É., Lee Y.-H., Goto Y., Réfrégiers M., Kardos J. (2018). BeStSel: A web server for accurate protein secondary structure prediction and fold recognition from the circular dichroism spectra. Nucleic Acids Res..

[40] Shih P., Holland D.R., Kirsch J.F. (1995). Thermal stability determinants of chicken egg-white lysozyme core mutants: Hydrophobicity, packing volume, and conserved buried water molecules. Protein Sci..

[41] Boothby T.C., Pielak G.J. (2017). Intrinsically Disordered Proteins and Desiccation Tolerance: Elucidating Functional and Mechanistic Underpinnings of Anhydrobiosis. BioEssays.

[42] Tompa P., Kovacs D. (2010). Intrinsically disordered chaperones in plants and animals. This paper is one of a selection of papers published in this special issue entitled “Canadian Society of Biochemistry, Molecular & Cellular Biology 52nd Annual Meeting—Protein Folding: Principles and Diseases” and has undergone the Journal’s usual peer review process. Biochem. Cell Biol..

[43] Lahtvee P.-J., Sánchez B.J., Smialowska A., Kasvandik S., Elsemman I.E., Gatto F., Nielsen J. (2017). Absolute Quantification of Protein and mRNA Abundances Demonstrate Variability in Gene-Specific Translation Efficiency in Yeast. Cell Syst..

[44] Ito Y., Selenko P. (2010). Cellular structural biology. Curr. Opin. Struct. Biol..

[45] Binolfi A., Theillet F., Selenko P. (2012). Bacterial in-cell NMR of human α-synuclein: A disordered monomer by nature?. Biochem. Soc. Trans..

[46] Croke R.L., Sallum C.O., Watson E., Watt E.D., Alexandrescu A.T. (2008). Hydrogen exchange of monomeric α-synuclein shows unfolded structure persists at physiological temperature and is independent of molecular crowding in Escherichia coli. Protein Sci..

[47] Van Der Lee R., Buljan M., Lang B., Weatheritt R.J., Daughdrill G.W., Dunker A.K., Fuxreiter M., Gough J., Gsponer J., Jones D.T. (2014). Classification of Intrinsically Disordered Regions and Proteins. Chem. Rev..

[48] Theillet F.-X., Binolfi A., Frembgen-Kesner T., Hingorani K., Sarkar M., Kyne C., Li C., Crowley P.B., Gierasch L., Pielak G.J. (2014). Physicochemical Properties of Cells and Their Effects on Intrinsically Disordered Proteins (IDPs). Chem. Rev..

[49] Cedeno C., Raveh-Hamit H., Dinnyes A., Tompa P. (2015). Towards Understanding Protein Disorder In-Cell. Adv. Exp. Med. Biol..

[50] Barbieri L., Luchinat E., Banci L. (2015). Protein interaction patterns in different cellular environments are revealed by in-cell NMR. Sci. Rep..

[51] Volkmer B., Heinemann M. (2011). Condition-Dependent Cell Volume and Concentration of Escherichia coli to Facilitate Data Conversion for Systems Biology Modeling. PLoS ONE.

[52] Atkinson J., Clarke M.W., Warnica J.M., Boddington K.F., Graether S.P. (2016). Structure of an Intrinsically Disordered Stress Protein Alone and Bound to a Membrane Surface. Biophys. J..

[53] Hanin M., Brini F., Ebel C., Toda Y., Takeda S., Masmoudi K. (2011). Plant dehydrins and stress tolerance. Plant Signal. Behav..

[54] Tompa P. (2002). Intrinsically unstructured proteins. Trends Biochem. Sci..

[55] Drira M., Saibi W., Brini F., Gargouri A., Masmoudi K., Hanin M. (2012). The K-Segments of the Wheat Dehydrin DHN-5 are Essential for the Protection of Lactate Dehydrogenase and β-Glucosidase Activities In Vitro. Mol. Biotechnol..

[56] Liu Y., Li D., Song Q., Zhang T., Li D., Yang X. (2019). The maize late embryogenesis abundant protein ZmDHN13 positively regulates copper tolerance in transgenic yeast and tobacco. Crop J..

[57] Yang W., Zhang L., Lv H., Li H., Zhang Y., Xu Y., Yu J. (2015). The K-segments of wheat dehydrin WZY2 are essential for its protective functions under temperature stress. Front Plant. Sci..

[58] Zilberstein D., Agmon V., Schuldiner S., Padan E. (1984). Escherichia coli intracellular pH, membrane potential, and cell growth. J. Bacteriol..

[59] Dosztányi Z., Csizmok V., Tompa P., Simon I. (2005). IUPred: Web server for the prediction of intrinsically unstructured regions of proteins based on estimated energy content. Bioinformatics.

[60] Szász C., Alexa A., Tóth K., Rakacs M., Langowski J., Tompa P. (2011). Protein Disorder Prevails under Crowded Conditions. Biochemistry.

[61] Banks A., Qin S., Weiss K.L., Stanley C.B., Zhou H.-X. (2018). Intrinsically Disordered Protein Exhibits Both Compaction and Expansion under Macromolecular Crowding. Biophys. J..

[62] Cino E.A., Karttunen M., Choy W.-Y. (2012). Effects of Molecular Crowding on the Dynamics of Intrinsically Disordered Proteins. PLoS ONE.

[63] Plitzko J.M., Schuler B., Selenko P. (2017). Structural Biology outside the box—inside the cell. Curr. Opin. Struct. Biol..

[64] Reiersen H., Rees A.R. (2000). Trifluoroethanol may form a solvent matrix for assisted hydrophobic interactions between peptide side chains. Protein Eng..

[65] Hamdi K., Salladini E., O’Brien D.P., Brier S., Chenal A., Yacoubi I., Longhi S. (2017). Structural disorder and induced folding within two cereal, ABA stress and ripening (ASR) proteins. Sci. Rep..

[66] Smith A.L., Dohn M.R., Brown M.V., Reynolds A.B. (2012). Association of Rho-associated protein kinase 1 with E-cadherin complexes is mediated by p120-catenin. Mol. Biol. Cell.

[67] McClellan A.J., Xia Y., Deutschbauer A.M., Davis R.W., Gerstein M., Frydman J. (2007). Diverse Cellular Functions of the Hsp90 Molecular Chaperone Uncovered Using Systems Approaches. Cell.

[68] Houry W.A., Frishman D., Eckerskorn C., Lottspeich F., Hartl F.U. (1999). Identification of in vivo substrates of the chaperonin GroEL. Nature.

[69] Cedeño C., Pauwels K., Tompa P. (2017). Protein Delivery into Plant Cells: Toward In vivo Structural Biology. Front. Plant Sci..

[70] Kiss R., Kovacs D., Tompa P., Perczel A. (2008). Local Structural Preferences of Calpastatin, the Intrinsically Unstructured Protein Inhibitor of Calpain. Biochemistry.

[71] Tompa P., Prilusky J., Silman I., Sussman J. (2008). Structural disorder serves as a weak signal for intracellular protein degradation. Proteins Struct. Funct. Bioinform..

[72] Gsponer J., Futschik M.E., Teichmann S.A., Babu M.M. (2008). Tight Regulation of Unstructured Proteins: From Transcript Synthesis to Protein Degradation. Science.

[73] Hegyi H., Tompa P. (2008). Intrinsically Disordered Proteins Display No Preference for Chaperone Binding In Vivo. PLoS Comput. Biol..

[74] Kovacs D., Tompa P. (2012). Diverse functional manifestations of intrinsic structural disorder in molecular chaperones. Biochem. Soc. Trans..

[75] Tolleter D., Hincha D.K., Macherel D. (2010). A mitochondrial late embryogenesis abundant protein stabilizes model membranes in the dry state. Biochim. Biophys. Acta (BBA) Biomembr..

[76] Habchi J., Tompa P., Longhi S., Uversky V.N. (2014). Introducing Protein Intrinsic Disorder. Chem. Rev..

[77] Faloona G.R., Srere P.A. (1969). Escherichia coli citrate synthase. Purification and the effect of potassium on some properties. Biochemistry.

[78] Weydert C.J., Cullen J.J. (2009). Measurement of superoxide dismutase, catalase and glutathione peroxidase in cultured cells and tissue. Nat. Protoc..

[79] Hartl F.U., Bracher A., Hayer-Hartl M. (2011). Molecular chaperones in protein folding and proteostasis. Nature.

