# Supplementary material for: Interplay of Structural Disorder and Short Binding Elements in the Cellular Chaperone Function of Plant Dehydrin ERD14
Source: Cells. 2020 Aug 7;9(8):1856. doi: 10.3390/cells9081856 (PMC7465474; doi:10.3390/cells9081856)
Supplement: Supplementary file 1 [file cells-09-01856-s001.zip › Supplementary legends.docx]

**Supplementary Figure S1** *HSQC spectrum of WT ERD14 at different temperatures and pHs*

To assist the transfer of chemical shift values of ERD14 resonances observed *in vitro* to that *in vivo*, HSQC spectra have been recorded *in vitro* under various temperatures and pHs, as follows. (A) Overlay of HSQC spectra measured at constant pH (6.58), at temperatures: 277 K (blue), 282.5 K (green) and 288 K (red). (B) Overlay of HSQC spectra measured at constant temperature (277 K), at pH values: 6.58 (red), 6.94 (orange), 7.29 (yellow), 7.42 (green), 7.57 (light blue), and 7.70 (navy). (C) The resulting assignment of the final reference spectrum at 277 K and pH 7.70, deposited in the BMRB database (Accession Number 26636).

**Supplementary Figure S2** *HSQC spectrum of ERD14 under crowding conditions*

Overlay of ^1^H-^15^N HSQC spectra of purified WT ERD14 at increasing dextran concentrations: 0 (blue), 150 (cyan), 300 (lime) and 400 mg/ml (green) dextran. Most of the resonances (and in particular, several of those that disappear under *in vivo* conditions, cf. Figure 2A and Suppl. Table S3) are observed even in the presence of 400 mg/ml dextran, i.e. non-specific viscosity effects and/or exclusion by crowding does not cause signal disappearance reminiscent of the in-cell spectrum of ERD14 (Figure 2A).

**Supplementary Figure S3** *Disorder prediction of WT ERD14 and Full-Scr ERD14*

Sequence-based disorder predictor IUPred [[55]](https://paperpile.com/c/pcCHKe/eZqt) was used to assess the structural disorder of WT ERD14 and Full-Scr ERD14 (cf. Supplementary Table S1) offering no protection to *E. coli* against stress (Figure 1B). Predicted values above the threshold 0.5 suggest that both sequences are entirely disordered.

**Supplementary Figure S4** *ERD14 does not leak from E. coli cells during in-cell NMR experiment*

To prove that labeled ERD14 does not leak from *E. coli* cells during NMR data acquisition, two experiments have been carried out. Cells were collected before and after the in-cell NMR experiment, and pelleted by centrifugation (13000g x 2 min). (A) Overlay of the spectrum of the sample taken after 3 hours of induction (“in-cell spectrum” of ERD14, red) with the spectrum of its supernatant (blue): the empty spectrum of supernatant shows that the protein is within the cells during the entire measurement. The finally attained assignment of in-cell resonances is indicated (cf. Supplementary Table S3). (B) HSQC spectra of the cells (red) and their supernatant (blue) were also recorded before induction of expression. The empty spectrum shows the absence of ERD14 before induction. (C) Both the supernatant and cells were also run on SDS-PAGE: ERD14, as indicated by the arrows, is present in only the cells both before and after the experiment. Lanes 1-4: samples before measurement, lanes 6-9: samples after the in-cell measurement; Lanes 1, 6: cells before induction, lanes 2, 7: supernatant before induction, lanes 3, 8: cells after 3h induction, lanes 4, 9: supernatant after 3h induction; lanes 5, 10: protein markers, with molecular weights as indicated (in kDa).

**Supplementary Figure S5** *HSQC spectrum of ERD14 in the presence of increasing TFE concentration*

Overlay of ^1^H-^15^N HSQC spectra of purified WT ERD14 at increasing TFE concentrations: 0% (purple), 5% (magenta), 10% (violet), 20% (cyan) and 30% (light blue) TFE. Due to change in the secondary structure propensity depending on the TFE content, peak shifting can be observed through the overlapped spectra, i.e. formation of secondary structure by TFE does not cause spectral changes similar to those observed in-cell spectrum of ERD14 (Figure 2A).

**Supplementary Figure S6** *In vitro protection of the cellular partners of ERD14*

(A) In vitro protection of catalase by 4x molar excess of ERD14 measured in a dehydration assay before (NS) and after (S) stress in the absence (light blue) and 4x excess (dark blue) of WT ERD14. (B) In vitro protection of citrate synthase by 20x molar excess of ERD14 under heat stress, as demonstrated by enzyme activity before (NS) and after (S) heat stress, in the absence (light blue) and 20x excess (dark blue) of WT ERD14. On both panels, data represent mean ± SEM.

**Supplementary Figure S7** *Growth curves of E. coli cells expressing different constructs*

The growth rate of different *E. coli* cultures was recorded by following the absorbance of the culture at 600 nm. Grey: cells without transformation, red: cells expressing WT ERD14, black: cells expressing calpastatin, blue: cells expressing Full-Scr ERD14. The initial lag-phase is dependent on the number of cells surviving transformation.

**Supplementary Table S1.** *Sequences of ERD14 constructs and controls*

(A) Deletion mutants of ERD14 are aligned to the sequence of WT ERD14, and the conserved segments are highlighted in color. (B) Full sequence of the scrambled mutant. (C) Sequences of GST and calpastatin used as controls in the cell protection experiments.

**Supplementary Table S2** *In-cell NMR results*

Analysis of the in-cell NMR spectrum of ERD14: raw results (i.e. observed changes compared to the reference) from the in-cell NMR spectrum (A) and from the dextran control spectrum (B) are listed. Residues for which no certain information is available are left out. The final, corrected results of the in-cell NMR spectrum are derived from the differences in (A) and (B), and are qualitatively marked by words (C) and quantitatively numbers (D). For comparison, conserved segments (E) are indicated as well as the in-cell ^15^N and ^1^H chemical shifts of the assigned (“steady”) resonances of ERD14 (F, G).

**Supplementary Table S3** *Protein partners of ERD14 identified by cross-linking and mass spectrometry*

UniProt Accession Code, Gene name and Protein name for all proteins bound to ERD14 as identified by MSMS, together with their peptide spectrum match (PSM) values in nonstressed

and stressed samples. Their abundance in E. coli (from PaxDb (Wang et al., 2015)), the normalized PSM values (relative to their abundance) and the ratio of stressed/non-stressed PSM values (factor of change) are also given.

Proteins above the first separation line have a significantly increased interaction potential (at least 2x more PSM in the stressed sample relative to the non-stressed sample, termed IIP), while proteins below the second separation line have a significantly decreased interaction potential (2x less PSM in the stressed sample relative to the non-stressed sample, termed DIP).

**Supplementary Table S4** *Protein partners of ERD14 significantly changing upon heat stress*

Proteins that significantly increase (IIP) or decrease (DIP) binding to ERD14 upon stress, identified by MSMS (cf. Experimental Procedures). Full list of ERD14 intercators is given in Suppl. Table S4, here the top 20 IIPs and top 10 DIPs are listed, along with their UniProt Accession Code, Gene name, Protein name, factor of change (between no stress (NS) and stress (S) conditions) and isoelectric point.

**Supplementary Table S5** *E. coli viability measurements*

Survival rates of E. coli cells overexpressing a variety of constructs following heat stress (50oC x 15 min. The protein overexpressed in the cell, number of independent experiments, mean, SD and SEM values of cell viability are shown.
