# Supplementary material for: Interplay of Structural Disorder and Short Binding Elements in the Cellular Chaperone Function of Plant Dehydrin ERD14
Source: Cells. 2020 Aug 7;9(8):1856. doi: 10.3390/cells9081856 (PMC7465474; doi:10.3390/cells9081856)
Supplement: Supplementary file 1 [file cells-09-01856-s001.zip › Supplementary Methods.docx]

***Experimental Procedures***

*In-cell NMR and spectral assignment*

BL21 (DE3) Star pLysS cells containing the wild type or the Full-Scr ERD14 construct were grown in LB medium (Sigma-Aldrich L3522) containing 50 mg/ml carbenicillin to an OD_600_ of about 0.6-0.8. Cells were collected by gentle centrifugation (under sterile conditions, 2700xg, 20 minutes, room temperature) and resuspended in fresh ^15^N-labeled M9 minimal medium (using a quarter of the initial volume of full medium). The M9 minimal medium contained 32 mM Na_2_HPO_4_, 22 mM KH_2_PO_4_, 8.6 mM NaCl, 0.1 mM CaCl_2_, 1 mM MgSO_4_, 50 mg/L carbenicillin, 10 mL/L vitamin mix from Sigma-Aldrich, 10 mL/L 100X trace elements solution, 1 g/L ^15^NH_4_Cl, 4 g/L glucose**^.^**H_2_O. The 100X trace elements solution consisted of 17 mM EDTA, 3 mM FeCl_3_, 0.6 mM ZnCl_2_, 0.08 mM CuCl_2_, 0.04 mM CoCl_2_, 0.16 mM H_3_BO_3_, 0.007 mM MnCl_2_. After one hour of additional growth at 37°C, cells were induced with 1.6 mM IPTG at 37 °C. Samples were taken before and after 3 hours of induction, respectively.

For both samples, 100 ml of bacterial culture were pelleted by gentle centrifugation (under sterile conditions, 3000xg, 20 min, 20 °C), resuspended in 1.5 mL of NMR-M9 medium (32 mM Na_2_HPO_4_, 22 mM KH_2_PO_4_, 8.6 mM NaCl, 0.1 mM CaCl_2_, 1 mM MgSO_4_, 50 mg/L carbenicillin, 4 g/L glucose**^.^**H_2_O), followed by another gentle centrifugation step and resuspension in 800 mL NMR-M9 buffer. Finally, 100 mL of D_2_O were added to 1 mL of this very dense cell suspension and inserted into a standard NMR tube. For both samples 1D (watergate), as well as 2D (^15^N-HSQC) in-cell NMR spectra were acquired on a Bruker DRX 500 MHz spectrometer at 277 K. HSQC spectra were recorded with a resolution of 1024 and 256 measured time points and spectral widths of 4.691 +/- 5 ppm and 118.49 +/- 11.5 ppm in the ^1^H and ^15^N dimensions, respectively, with 4/8 (no induction) and 4/16 (induced) dummy/regular scans.

Cell suspensions were recollected from the NMR tube right after the in-cell NMR measurements, and pelleted by centrifugation (13000g x 2 min, RT). DSS was added, and the spectrum of the supernatant was acquired in exactly the same way as for the in-cell spectra, so as to give evidence of the true in-cell status of the measured protein (no leaking from the cells). Furthermore, at all stages of the in-cell experiment (in-cell spectra and supernatant spectra, before and after the measurements) samples were taken for gel electrophoresis and SDS-PAGE was performed (Suppl. Figure S4C).

*In vitro references and controls to the in-cell NMR experiments*

NMR peaks assigned *in vitro* (181/185^30^) were transferred to the *in vivo* state by taking into consideration that the intracellular pH of bacterial cells is relatively high [[54]](https://paperpile.com/c/pcCHKe/snWd). To this end, WT and Full-Scr ERD14 were expressed and purified as described previously [[31]](https://paperpile.com/c/pcCHKe/Rt6E). Lyophilized, ^15^N‑labeled ERD14 was dissolved at a concentration of about 1 mM in 550 ml of NMR-M9 buffer containing 10% D_2_O and 1 mM DSS. *In vitro* HSQC spectra were recorded at 277 K at several pH values (6.58, 6.94, 7.29, 7.42, 7.57, 7.70), and at pH 6.58 at different temperatures (288 K, 282.5 K and 277 K) to transfer the previous assignment to the temperature and pH conditions of the in-cell spectrum (Suppl. Figure S1A and S1B, respectively). WT ERD14 chemical shifts have been deposited to the BioMagResBank under BMRB Accession Number 26636). The HSQC spectrum at 277 K and pH 7.7 was then used as a reference for the in-cell spectra (Suppl. Figure S1C, Suppl. Table S2).

In order to mimic non-specific crowding effects within the cell, different amounts of dextran (from Leuconostoc ssp, Fluka, MW ~70 kDa) were added to reach final concentrations of 0, 150, 300 and 400 mg/ml, and HSQC spectra were recorded at pH 7.79 and 277 K (Suppl. Figure S2). The same sample was used as in the temperature- and pH-titration. A small pH shift of 7.79 to 7.63 was observed (but not corrected for). All listed experiments were acquired on a Bruker DRX 500 MHz spectrometer using the same parameters as for the in-cell spectra, unless stated otherwise.

*Interpretation of in cell NMR spectra*

For the interpretation of the in-cell results, the observed differences between in-cell and reference spectra were categorized. We marked resonances “steady” if they are present in both spectra in the same position with high confidence level, “disappearing”, if they are missing from the in-cell spectrum, “broadening”, if they are detectable but because of poor S/N ratio and severe signal overlap within the cell, they are hard to unequivocally assign in the cell. A small number of resonances, the presence of which could not be ascertained, are marked as “unknown” (all marks are given in Suppl. Table S3). For the interpretation of results, i.e. to see if changes cluster to certain regions and correlate with structural-functional elements of ERD14, we visualized these scores by giving -1 for disappearing, 0 for broadening and +1 for steady residues, taking into account only residues which were not disappearing in the presence of 400 g/l dextran (Figure 2F).

*Determination of absolute protein concentration in the cell*

In order to ascertain that we can detect the whole amount of expressed WT ERD14 in cells, which is responsible for the function *in vivo*, and to determine the protein concentration within the cells, we conducted the following experiment based on [[37,74]](https://paperpile.com/c/pcCHKe/RKhG+7haO). Our in-cell samples, on the average, contained 8 ± 0.96 x 10^10^ cells (calculated as given in [[75]](https://paperpile.com/c/pcCHKe/YrVJ)). To prepare lysates of in-cell NMR samples, cells were sedimented for at 3000g x 20 min and resuspended in NMR-M9 buffer. After sonication and boiling for 5 min, denatured globular proteins were sedimented by centrifugation for 12100 rpm x 30 min. The cell extract was purified on ResourceQ anion exchange column, dialyzed into MQ water and lyophilized. The purified protein was dissolved into NMR-M9 buffer and transferred to the original NMR tubes. 1D/2D NMR spectra were recorded with identical spectrometer and acquisition settings as for the intact in-cell NMR samples. The quantity and intactness of WT ERD14 and Full-Scr ERD14 were analyzed on SDS-PAGE gel. Comparing the obtained peak intensity ratios (Figure 2B), the 1D signal ratios of in-cell and purified protein NMR spectra and the band intensities on SDS-PAGE gels, the protein concentration was calculated based on [[37, 76]](https://paperpile.com/c/pcCHKe/TP7T). We found that the cells contained 177 ± 30 µM WT ERD14 protein during the in-cell NMR measurements, which is in the physiological range of ERD14, as dehydrin amount can reach 4% of the proteome under stress conditions [[77]](https://paperpile.com/c/pcCHKe/TP7T). The bacterial cytoplasm contains 300 mg/ml proteins which is about 25% of the total cell volume [[64]](https://paperpile.com/c/pcCHKe/TP7T). In this case, 4% of the proteome is 12 mg/ml. In our NMR and cell viability experiments, the cells contained about 3.7 mg/ml ERD14 protein, which is approximately 3-times less than its maximum concentration attained upon stress.

*Protein stability in the cell*

BL21 star cells transformed with ERD14/pT7-FLAG 2 construct were grown in LB medium containing 50 mg/ml carbenicillin at 37 °C, 250 rpm up to an OD_600_ of 0,5-0,6. Expression of FLAG-tagged ERD14 was induced by 0.5 mM IPTG at 37°C for 30 minutes. Cells were collected by gentle centrifugation (3600 rpm x 6 min) and resuspended in fresh NZYM without IPTG. Cells were further incubated at 37 °C, 250 rpm for an additional 24 hours. To detect half-life of ERD14 after heat stress, the resuspended cells were stressed at 50 °C for 15 min, 1 ml samples were taken at the indicated time points (0, 1, 2, 3, 4, 5, 6, 24h), centrifuged and resuspended in SDS loading buffer, boiled, applied to 15% SDS-PAGE and blotted to a nitrocellulose membrane with the BioRad Trans-Blot Turbo Transfer System. For immunodetection, monoclonal Anti-FLAG M2 antibody (Sigma F1804) and ECL Anti-mouse IgG HRP conjugate secondary antibody (GE NA931V) were used in SNAP i.d. 2.0 device (Millipore). To develop the membrane, Amersham ECL Prime substrate was used by exposing to X-ray film (Primax). The same experiment was carried out using pGEX-5X 1 vector for the expression of the control GST protein. For detection, monoclonal Anti-GST antibody (Sigma G1160) was used with the same secondary antibody.

*Citrate synthase activity assay*

The chaperone activity of ERD14 was measured in vitro using temperature deactivation of citrate synthase (EC 2.3.3.1 from Megazyme). The assay is a modified protocol based on the method described by Faloona and Srere [[78]](https://paperpile.com/c/pcCHKe/RL4i). Citrate synthase converts Acetyl-CoA (A2056, Sigma) and oxaloacetate (O4126, Sigma) into citrate and reduced coenzyme-A. The latter reacts with Ellman’s reagent (DTNB, D8130 from, Sigma). The reaction was followed for 5 min at 412 nm in a microplate in Biotek Synergy Mx Plate-reader, in an assay containing at final concentrations 6 nM citrate synthase, 0.45 mM Acetyl-coA, 0.5 mM oxaloacetate and 0.1 mM DTNB, in a buffer of 50 mM Tris-HCl, pH 8. The activity of the enzyme was measured before stress and after 15 min at 50 °C, in the absence and 20x excess of ERD14. The initial activity of the control enzymatic reaction was taken as 100%.

*Catalase activity assay*

0.8 M catalase was treated in the absence and presence of 3.2 μM ERD14. The samples were then dehydrated overnight at RT in a vacuum chamber with silica gel. The same amount of MiliQ water as the evaporated buffer was added to rehydrate the samples 40 min and 60 min prior to the measurement. Catalase activity was performed as described by Weydert and Cullen [79], and measured based on the peroxide removal by BioTek Synegy plate reader at 240 nm and 25°C.
