# Supplementary material for: Interplay of Structural Disorder and Short Binding Elements in the Cellular Chaperone Function of Plant Dehydrin ERD14
Source: Cells. 2020 Aug 7;9(8):1856. doi: 10.3390/cells9081856 (PMC7465474; doi:10.3390/cells9081856)
Supplement: Supplementary file 1 [file cells-09-01856-s001.zip › Supplementary tables_revised.docx]

**Table S1.**

**A. Wild type ERD14**

Ka segment

**ERD14 wt** MAEEIKNVPEQEVPKVATEESSAEVTDRGLFDFLGKKKDETKPEETPIASEFEQKVHISEPE

**S segment ChP segment Kb segment**

PEVKHESLLEKLHRSDSSSSSSSEEEGSDGEKRKKKKEKKKPTTEVEVKEEEKKGFMEKLKE

**H region Kc segment**

KLPGHKKPEDGSAVAAAPVVVPPPVEEAHPVEKKGILEKIKEKLPGYHPKTTVEEEKKDKE

**B. Full Scr-ERD14:**

**Full-Scr** MKTGKLPEETSAFEKSNESIVGETKEDKMEESPEPIVSKSKVEEQEEKKEKSTEALEVKPLP

EEYASRVEPGPHAEVFDGEKVRHGGEEPGFLPAHGKSSVKTEKESAIEVRHETTEPELVEQK

KLKPPIHKPKEDLEDVEHKEGVVDKKTLPVKDLSKIKSEKMKDSEAVPKAKSKEKEPKKFEA

**C. Control proteins:**

**GST** MSPILGYWKIKGLVQPTRLLLEYLEEKYEEHLYERDEGDKWRNKKFELGLEFPNLPYYIDGD

VKLTQSMAIIRYIADKHNMLGGCPKERAEISMLEGAVLDIRYGVSRIAYSKDFETLKVDFLS

KLPEMLKMFEDRLCHKTYLNGDHVTHPDFMLYDALDVVLYMDPMCLDAFPKLVCFKKRIEAI

PQIDKYLKSSKYIAWPLQGWQATFGGGDHPPKSDLIEGRGIPEFPGRLERPHRD

**Calpastatin**

MASHHHHHHGDPTETKAIPVSQQMEGPHLPNKKKHKKQAVKTEPEKKSQSTKLSVVHEKKSQ

EGKPKEHTEPKSLPKQASDTGSNDAHNKKAVSRSAEQQPSEKSTEPKTKPQDMISAGGESVA

GITAISGKPGDKKKEKKSLTPAVPVESKPDKPSGKSGMDAALDDLIDTLGGPEETEEENTTY

TGPEVSDPMSSTYIEELGKREVTIPPKYRELLAKKEGITGPPADSSKPIGPDDAIDALSSDF

TCGSPTAAGKKTEKEESTEVLKAQSAGTVRSAAPPQEKKRKVEKDTMSDQALEALSASLGTR

QAEPELDLRSIKEVDEAKAKEEKLEKCGEDDETIPSEYRLKPATDKDGKPLLPEPEEKPKPR

SESELIDELSEDFDRSECKEKPSKPTEKTEESKAAAPAHHHHHH

**Table S2**

| **Residue number** | **name** | **in-cell NMR: raw data** | **Control experiment: dextrane** | **in-cell NMR: final result** | **Final dis­apperance score:** | **Conserved segments** | **in-cell ^15^N chem. shift** | **in-cell ^1^H chem. shift** |
| --- | --- | --- | --- | --- | --- | --- | --- | --- |
|  |  | **(A)** | **(B)** | **(C)** | **(D)** | **(E)** | **(F)** | **(G)** |
| **1** | **Met** |  |  | unknown | 0 |  |  |  |
| **2** | **Ala** |  |  | unknown | 0 |  |  |  |
| **3** | **Glu** |  |  | unknown | 0 |  |  |  |
| **4** | **Glu** | steady |  | steady | 1 |  | 123.87 | 8.767 |
| **5** | **Ile** | steady |  | steady | 1 |  | 124.61 | 8.553 |
| **6** | **Lys** | steady |  | steady | 1 |  | 126.99 | 8.621 |
| **7** | **Asn** | steady |  | steady | 1 |  | 121.06 | 8.645 |
| **8** | **Val** |  |  | broadening | 0 |  |  |  |
| **9** | **Pro** |  |  | unknown | 0 |  |  |  |
| **10** | **Glu** | steady |  | steady | 1 |  | 121.59 | 8.689 |
| **11** | **Gln** |  |  | broadening | 0 |  |  |  |
| **12** | **Glu** |  |  | broadening | 0 |  |  |  |
| **13** | **Val** | steady |  | steady | 1 |  | 124.59 | 8.471 |
| **14** | **Pro** |  |  | unknown | 0 |  |  |  |
| **15** | **Lys** |  |  | broadening | 0 |  |  |  |
| **16** | **Val** |  |  | unknown | 0 |  |  |  |
| **17** | **Ala** | steady |  | steady | 1 |  | 129.17 | 8.662 |
| **18** | **Thr** | steady |  | steady | 1 |  | 114.52 | 8.321 |
| **19** | **Glu** |  |  | broadening | 0 |  |  |  |
| **20** | **Glu** |  |  | broadening | 0 |  |  |  |
| **21** | **Ser** | steady |  | steady | 1 |  | 117.64 | 8.562 |
| **22** | **Ser** | steady |  | steady | 1 |  | 118.51 | 8.529 |
| **23** | **Ala** |  |  | broadening | 0 |  |  |  |
| **24** | **Glu** | steady |  | steady | 1 | Ka | 120.36 | 8.422 |
| **25** | **Val** |  |  | unknown | 0 | Ka |  |  |
| **26** | **Thr** |  |  | broadening | 1 | Ka |  |  |
| **27** | **Asp** |  |  | broadening | 0 | Ka |  |  |
| **28** | **Arg** |  |  | broadening | 0 | Ka |  |  |
| **29** | **Gly** | disappearing |  | disappearing | -1 | Ka |  |  |
| **30** | **Leu** | disappearing |  | disappearing | -1 | Ka |  |  |
| **31** | **Phe** | disappearing | disappearing | disappearing | -1 | Ka |  |  |
| **32** | **Asp** |  |  | broadening | 0 | Ka |  |  |
| **33** | **Phe** | disappearing |  | disappearing | -1 | Ka |  |  |
| **34** | **Leu** |  |  | broadening | 0 | Ka |  |  |
| **35** | **Gly** | disappearing |  | disappearing | -1 | Ka |  |  |
| **36** | **Lys** | disappearing |  | disappearing | -1 | Ka |  |  |
| **37** | **Lys** |  |  | broadening | 0 | Ka |  |  |
| **38** | **Lys** |  |  | broadening | 0 | Ka |  |  |
| **39** | **Asp** |  |  | broadening | 0 | Ka |  |  |
| **40** | **Glu** |  |  | broadening | 0 | Ka |  |  |
| **41** | **Thr** | steady |  | steady | 1 | Ka | 116.32 | 8.440 |
| **42** | **Lys** |  |  | broadening | 0 | Ka |  |  |
| **43** | **Pro** |  |  | unknown | 0 | Ka |  |  |
| **44** | **Glu** | steady |  | steady | 1 |  | 121.56 | 8.755 |
| **45** | **Glu** |  |  | broadening | 0 |  |  |  |
| **46** | **Thr** | steady |  | steady | 1 |  | 119.68 | 8.479 |
| **47** | **Pro** |  |  | unknown | 0 |  |  |  |
| **48** | **Ile** |  |  | broadening | 0 |  |  |  |
| **49** | **Ala** | steady |  | steady | 1 |  | 129.34 | 8.571 |
| **50** | **Ser** | steady |  | steady | 1 |  | 116.12 | 8.479 |
| **51** | **Glu** |  |  | broadening | 0 |  |  |  |
| **52** | **Phe** | steady |  | steady | 1 |  | 120.17 | 8.231 |
| **53** | **Glu** |  |  | broadening | 0 |  |  |  |
| **54** | **Gln** |  |  | broadening | 0 |  |  |  |
| **55** | **Lys** |  |  | broadening | 0 |  |  |  |
| **56** | **Val** |  |  | broadening | 0 |  |  |  |
| **57** | **His** | disappearing |  | disappearing | -1 |  |  |  |
| **58** | **Ile** | disappearing | disappearing | disappearing | -1 |  |  |  |
| **59** | **Ser** |  |  | broadening | 0 |  |  |  |
| **60** | **Glu** | steady |  | steady | 1 |  | 124.99 | 8.589 |
| **61** | **Pro** |  |  | unknown | 0 |  |  |  |
| **62** | **Glu** |  |  | broadening | 0 |  |  |  |
| **63** | **Pro** |  |  | unknown | 0 |  |  |  |
| **64** | **Glu** | steady |  | steady | 1 |  | 122.27 | 8.657 |
| **65** | **Val** |  |  | broadening | 0 |  |  |  |
| **66** | **Lys** | disappearing | disappearing | disappearing | -1 |  |  |  |
| **67** | **His** |  |  | broadening | 0 |  |  |  |
| **68** | **Glu** |  |  | broadening | 0 |  |  |  |
| **69** | **Ser** | disappearing | disappearing | disappearing | -1 |  |  |  |
| **70** | **Leu** |  |  | broadening | 0 |  |  |  |
| **71** | **Leu** | disappearing |  | disappearing | -1 |  |  |  |
| **72** | **Glu** |  |  | broadening | 0 |  |  |  |
| **73** | **Lys** |  |  | broadening | 0 |  |  |  |
| **74** | **Leu** |  |  | broadening | 0 | S |  |  |
| **75** | **His** |  |  | broadening | 1 | S |  |  |
| **76** | **Arg** |  |  | broadening | 0 | S |  |  |
| **77** | **Ser** |  |  | broadening | 1 | S |  |  |
| **78** | **Asp** |  |  | broadening | 0 | S |  |  |
| **79** | **Ser** |  |  | broadening | 1 | S |  |  |
| **80** | **Ser** | steady |  | steady | 1 | S | 118.25 | 8.525 |
| **81** | **Ser** |  |  | broadening | 1 | S |  |  |
| **82** | **Ser** | steady |  | steady | 1 | S | 118.16 | 8.479 |
| **83** | **Ser** | steady |  | steady | 1 | S | 118.22 | 8.503 |
| **84** | **Ser** | steady |  | steady | 1 | S | 118.22 | 8.503 |
| **85** | **Ser** | steady |  | steady | 1 | S | 118.22 | 8.503 |
| **86** | **Glu** |  |  | broadening | 0 |  |  |  |
| **87** | **Glu** | steady |  | steady | 1 | ChP | 121.62 | 8.481 |
| **88** | **Glu** |  |  | broadening | 0 | ChP |  |  |
| **89** | **Gly** | steady |  | steady | 1 | ChP | 110.44 | 8.614 |
| **90** | **Ser** | steady |  | steady | 1 | ChP | 116.10 | 8.448 |
| **91** | **Asp** | steady |  | steady | 1 | ChP | 122.31 | 8.688 |
| **92** | **Gly** | steady |  | steady | 1 | ChP | 109.04 | 8.454 |
| **93** | **Glu** |  |  | broadening | 0 | ChP |  |  |
| **94** | **Lys** |  |  | broadening | 0 | ChP |  |  |
| **95** | **Arg** |  |  | broadening | 0 | ChP |  |  |
| **96** | **Lys** |  |  | broadening | 0 | ChP |  |  |
| **97** | **Lys** |  |  | broadening | 0 | ChP |  |  |
| **98** | **Lys** |  |  | broadening | 0 | ChP |  |  |
| **99** | **Lys** |  |  | broadening | 0 | ChP |  |  |
| **100** | **Glu** |  |  | broadening | 0 | ChP |  |  |
| **101** | **Lys** |  |  | broadening | 0 | ChP |  |  |
| **102** | **Lys** |  |  | broadening | 0 | ChP |  |  |
| **103** | **Lys** |  |  | broadening | 0 | ChP |  |  |
| **104** | **Pro** |  |  | unknown | 0 |  |  |  |
| **105** | **Thr** |  |  | broadening | 0 |  |  |  |
| **106** | **Thr** | steady |  | steady | 1 |  | 116.53 | 8.298 |
| **107** | **Glu** |  |  | broadening | 0 |  |  |  |
| **108** | **Val** |  |  | broadening | 0 |  |  |  |
| **109** | **Glu** | disappearing | disappearing | broadening | 0 |  |  |  |
| **110** | **Val** |  |  | broadening | 0 |  |  |  |
| **111** | **Lys** | disappearing | disappearing | broadening | 0 |  |  |  |
| **112** | **Glu** |  |  | broadening | 0 |  |  |  |
| **113** | **Glu** |  |  | broadening | 0 | Kb |  |  |
| **114** | **Glu** |  |  | broadening | 0 | Kb |  |  |
| **115** | **Lys** |  |  | broadening | 0 | Kb |  |  |
| **116** | **Lys** |  |  | broadening | 0 | Kb |  |  |
| **117** | **Gly** | disappearing |  | disappearing | -1 | Kb |  |  |
| **118** | **Phe** | disappearing |  | disappearing | -1 | Kb |  |  |
| **119** | **Met** |  |  | broadening | 0 | Kb |  |  |
| **120** | **Glu** |  |  | broadening | 0 | Kb |  |  |
| **121** | **Lys** |  |  | broadening | 0 | Kb |  |  |
| **122** | **Leu** | disappearing | disappearing | disappearing | -1 | Kb |  |  |
| **123** | **Lys** |  |  | broadening | 0 | Kb |  |  |
| **124** | **Glu** |  |  | broadening | 0 | Kb |  |  |
| **125** | **Lys** |  |  | broadening | 0 | Kb |  |  |
| **126** | **Leu** | disappearing |  | disappearing | -1 | Kb |  |  |
| **127** | **Pro** |  |  | unknown | 0 | Kb |  |  |
| **128** | **Gly** | disappearing |  | disappearing | -1 | Kb |  |  |
| **129** | **His** | disappearing |  | disappearing | -1 | Kb |  |  |
| **130** | **Lys** | disappearing |  | disappearing | -1 | Kb |  |  |
| **131** | **Lys** |  |  | disappearing | -1 | Kb |  |  |
| **132** | **Pro** |  |  | unknown | 0 | Kb |  |  |
| **133** | **Glu** | steady |  | steady | 1 | Kb | 121.49 | 8.842 |
| **134** | **Asp** |  |  | broadening | 0 | Kb |  |  |
| **135** | **Gly** | steady |  | steady | 1 |  | 110.37 | 8.550 |
| **136** | **Ser** | steady |  | steady | 1 |  | 116.15 | 8.313 |
| **137** | **Ala** | steady |  | steady | 1 |  | 126.06 | 8.425 |
| **138** | **Val** | steady |  | steady | 1 |  | 120.13 | 8.192 |
| **139** | **Ala** | steady |  | steady | 1 |  | 128.79 | 8.485 |
| **140** | **Ala** |  |  | broadening | 0 |  |  |  |
| **141** | **Ala** | disappearing |  | disappearing | -1 |  |  |  |
| **142** | **Pro** |  |  | unknown | 0 |  |  |  |
| **143** | **Val** |  |  | broadening | 0 |  |  |  |
| **144** | **Val** | disappearing |  | disappearing | -1 |  |  |  |
| **145** | **Val** | steady |  | steady | 1 |  | 129.14 | 8.627 |
| **146** | **Pro** |  |  | unknown | 0 |  |  |  |
| **147** | **Pro** |  |  | unknown | 0 |  |  |  |
| **148** | **Pro** |  |  | unknown | 0 |  |  |  |
| **149** | **Val** |  |  | broadening | 0 |  |  |  |
| **150** | **Glu** |  |  | disappearing | -1 |  |  |  |
| **151** | **Glu** |  |  | broadening | 0 |  |  |  |
| **152** | **Ala** |  |  | broadening | 0 |  |  |  |
| **153** | **His** | disappearing |  | disappearing | -1 |  |  |  |
| **154** | **Pro** |  |  | unknown | 0 |  |  |  |
| **155** | **Val** |  |  | broadening | 0 |  |  |  |
| **156** | **Glu** |  |  | disappearing | -1 | Kc |  |  |
| **157** | **Lys** |  |  | broadening | 0 | Kc |  |  |
| **158** | **Lys** |  |  | broadening | 0 | Kc |  |  |
| **159** | **Gly** | steady |  | steady | 1 | Kc | 110.67 | 8.615 |
| **160** | **Ile** | disappearing |  | disappearing | -1 | Kc |  |  |
| **161** | **Leu** |  |  | broadening | 0 | Kc |  |  |
| **162** | **Glu** |  |  | broadening | 0 | Kc |  |  |
| **163** | **Lys** |  |  | broadening | 0 | Kc |  |  |
| **164** | **Ile** |  |  | broadening | 0 | Kc |  |  |
| **165** | **Lys** |  |  | broadening | 0 | Kc |  |  |
| **166** | **Glu** |  |  | broadening | 0 | Kc |  |  |
| **167** | **Lys** |  |  | broadening | 0 | Kc |  |  |
| **168** | **Leu** | disappearing |  | disappearing | -1 | Kc |  |  |
| **169** | **Pro** |  |  | unknown | 0 | Kc |  |  |
| **170** | **Gly** | disappearing |  | disappearing | -1 | Kc |  |  |
| **171** | **Tyr** | disappearing |  | disappearing | -1 |  |  |  |
| **172** | **His** | disappearing | disappearing | disappearing | -1 |  |  |  |
| **173** | **Pro** |  |  | unknown | 0 |  |  |  |
| **174** | **Lys** |  |  | broadening | 0 |  |  |  |
| **175** | **Thr** | steady |  | steady | 1 |  | 116.33 | 8.422 |
| **176** | **Thr** |  |  | broadening | 0 |  |  |  |
| **177** | **Val** |  |  | broadening | 0 |  |  |  |
| **178** | **Glu** |  |  | broadening | 0 |  |  |  |
| **179** | **Glu** |  |  | broadening | 0 |  |  |  |
| **180** | **Glu** |  |  | broadening | 0 |  |  |  |
| **181** | **Lys** |  |  | broadening | 0 |  |  |  |
| **182** | **Lys** |  |  | broadening | 0 |  |  |  |
| **183** | **Asp** | steady |  | steady | 1 |  | 121.64 | 8.509 |
| **184** | **Lys** | steady |  | steady | 1 |  | 121.36 | 8.302 |
| **185** | **Glu** | steady |  | steady | 1 |  | 127.57 | 8.166 |

**Table S3.**

| **UniProt AC** | **Gene name** | **Protein name** | **PSM in  non-stressed sample** | **PSM in stressed sample** | **E. coli abundance in ppm  (from PaxDB)** | **Normalized PSM non-stressed** | **Normalized PSM stressed** | **Factor of change (S/NS)** |
| --- | --- | --- | --- | --- | --- | --- | --- | --- |
| P33570 | tktB | Transketolase 2 - Escherichia coli - [TKT2_ECOLI] | 6 | 32 | 296.7 | 0.0202 | 0.1078 | 5.33 |
| P09152 | narG | Respiratory nitrate reductase 1 alpha chain - Escherichia coli - [NARG_ECOLI] | 16 | 69 | 57.6 | 0.2779 | 1.1984 | 4.31 |
| P21599 | pykA | Pyruvate kinase II - Escherichia coli - [KPYK2_ECOLI] | 11 | 46 | 619.9 | 0.0177 | 0.0742 | 4.18 |
| P07014 | sdhB | Succinate dehydrogenase iron-sulfur subunit - Escherichia coli - [DHSB_ECOLI] | 6 | 23 | 357.5 | 0.0168 | 0.0643 | 3.83 |
| P0A6L0 | deoC | Deoxyribose-phosphate aldolase - Escherichia coli - [DEOC_ECOLI] | 5 | 19 | 457.6 | 0.0109 | 0.0415 | 3.80 |
| P12758 | udp | Uridine phosphorylase - Escherichia coli - [UDP_ECOLI] | 11 | 40 | 2135.4 | 0.0052 | 0.0187 | 3.64 |
| P32176 | fdoG | Formate dehydrogenase-O. major subunit - Escherichia coli - [FDOG_ECOLI] | 10 | 36 | 22.3 | 0.4490 | 1.6164 | 3.60 |
| P0A8L1 | serS | Seryl-tRNA synthetase - Escherichia coli - [SYS_ECOLI] | 24 | 81 | 701.5 | 0.0342 | 0.1155 | 3.38 |
| P23839 | yicC | Protein yicC. - Escherichia coli - [YICC_ECOLI] | 6 | 19 | 108.6 | 0.0552 | 0.1749 | 3.17 |
| P0ABB4 | atpD | ATP synthase subunit beta - Escherichia coli - [ATPB_ECOLI] | 75 | 236 | 5972.2 | 0.0126 | 0.0395 | 3.15 |
| P33602 | nuoG | NADH-quinone oxidoreductase subunit G - Escherichia coli - [NUOG_ECOLI] | 23 | 69 | 123.7 | 0.1859 | 0.5578 | 3.00 |
| P61517 | can | Carbonic anhydrase 2 - Escherichia coli - [CAN_ECOLI] | 5 | 15 | 523.0 | 0.0096 | 0.0287 | 3.00 |
| P76558 | maeB | NADP-dependent malic enzyme - Escherichia coli - [MAO2_ECOLI] | 17 | 50 | 411.3 | 0.0413 | 0.1216 | 2.94 |
| P06959 | aceF | Dihydrolipoyllysine-residue acetyltransferase component of p - Escherichia coli - [ODP2_ECOLI] | 22 | 64 | 1848.2 | 0.0119 | 0.0346 | 2.91 |
| P0A9C5 | glnA | Glutamine synthetase - Escherichia coli - [GLNA_ECOLI] | 6 | 17 | 1714.6 | 0.0035 | 0.0099 | 2.83 |
| P31120 | glmM | Phosphoglucosamine mutase - Escherichia coli - [GLMM_ECOLI] | 11 | 31 | 529.9 | 0.0208 | 0.0585 | 2.82 |
| P75823 | ltaE | Low specificity L-threonine aldolase - Escherichia coli - [LTAE_ECOLI] | 5 | 14 | 93.8 | 0.0533 | 0.1493 | 2.80 |
| P02925 | rbsB | D-ribose-binding periplasmic protein - Escherichia coli - [RBSB_ECOLI] | 13 | 36 | 493.8 | 0.0263 | 0.0729 | 2.77 |
| P21179 | katE | Catalase HPII - Escherichia coli - [CATE_ECOLI] | 17 | 46 | 202.2 | 0.0841 | 0.2275 | 2.71 |
| P0ABA4 | atpH | ATP synthase delta chain - Escherichia coli - [ATPD_ECOLI] | 8 | 21 | 795.9 | 0.0101 | 0.0264 | 2.63 |
| P00864 | ppc | Phosphoenolpyruvate carboxylase - Escherichia coli - [CAPP_ECOLI] | 21 | 55 | 1177.8 | 0.0178 | 0.0467 | 2.62 |
| P30177 | ybiB | Uncharacterized protein ybiB. - Escherichia coli - [YBIB_ECOLI] | 5 | 13 | 193.7 | 0.0258 | 0.0671 | 2.60 |
| P00490 | malP | Maltodextrin phosphorylase - Escherichia coli - [PHSM_ECOLI] | 9 | 23 | 80.7 | 0.1116 | 0.2852 | 2.56 |
| P0ABB0 | atpA | ATP synthase subunit alpha - Escherichia coli - [ATPA_ECOLI] | 61 | 155 | 2627.9 | 0.0232 | 0.0590 | 2.54 |
| P15034 | pepP | Xaa-Pro aminopeptidase - Escherichia coli - [AMPP_ECOLI] | 8 | 20 | 331.0 | 0.0242 | 0.0604 | 2.50 |
| P0A796 | pfkA | 6-phosphofructokinase isozyme 1 - Escherichia coli - [K6PF1_ECOLI] | 19 | 47 | 999.9 | 0.0190 | 0.0470 | 2.47 |
| P0A6L4 | nanA | N-acetylneuraminate lyase - Escherichia coli - [NANA_ECOLI] | 7 | 17 | 29.3 | 0.2386 | 0.5795 | 2.43 |
| P31979 | nuoF | NADH-quinone oxidoreductase subunit F - Escherichia coli - [NUOF_ECOLI] | 7 | 17 | 306.8 | 0.0228 | 0.0554 | 2.43 |
| P0A6F3 | glpK | Glycerol kinase - Escherichia coli - [GLPK_ECOLI] | 40 | 96 | 835.0 | 0.0479 | 0.1150 | 2.40 |
| P0AB77 | kbl | 2-amino-3-ketobutyrate coenzyme A ligase - Escherichia coli - [KBL_ECOLI] | 15 | 36 | 241.9 | 0.0620 | 0.1488 | 2.40 |
| P0A9M2 | hpt | Hypoxanthine phosphoribosyltransferase - Escherichia coli - [HPRT_ECOLI] | 5 | 12 | 254.1 | 0.0197 | 0.0472 | 2.40 |
| P00956 | ileS | Isoleucyl-tRNA synthetase - Escherichia coli - [SYI_ECOLI] | 26 | 62 | 440.2 | 0.0591 | 0.1408 | 2.38 |
| P63284 | clpB | Chaperone protein clpB - Escherichia coli - [CLPB_ECOLI] | 157 | 373 | 1570.1 | 0.1000 | 0.2376 | 2.38 |
| P0A7A9 | ppa | Inorganic pyrophosphatase - Escherichia coli - [IPYR_ECOLI] | 8 | 19 | 2273.6 | 0.0035 | 0.0084 | 2.38 |
| P0A8T7 | rpoC | DNA-directed RNA polymerase subunit beta' - Escherichia coli - [RPOC_ECOLI] | 153 | 362 | 3754.9 | 0.0407 | 0.0964 | 2.37 |
| P13029 | katG | Peroxidase/catalase HPI - Escherichia coli - [CATA_ECOLI] | 28 | 66 | 526.1 | 0.0532 | 0.1255 | 2.36 |
| P0ADR8 | ygdH | Uncharacterized protein ygdH. - Escherichia coli - [YGDH_ECOLI] | 6 | 14 | 73.7 | 0.0814 | 0.1899 | 2.33 |
| P0AGD3 | sodB | Superoxide dismutase [Fe] - Escherichia coli - [SODF_ECOLI] | 13 | 30 | 1337.7 | 0.0097 | 0.0224 | 2.31 |
| P0A8M3 | thrS | Threonyl-tRNA synthetase - Escherichia coli - [SYT_ECOLI] | 20 | 46 | 1131.4 | 0.0177 | 0.0407 | 2.30 |
| P0C0S1 | mscS | Small-conductance mechanosensitive channel. - Escherichia coli - [MSCS_ECOLI] | 17 | 39 | 154.0 | 0.1104 | 0.2533 | 2.29 |
| P0AFG6 | sucB | Dihydrolipoyllysine-residue succinyltransferase component of - Escherichia coli - [ODO2_ECOLI] | 15 | 34 | 1086.2 | 0.0138 | 0.0313 | 2.27 |
| P0A9Q7 | adhE | Aldehyde-alcohol dehydrogenase - Escherichia coli - [ADHE_ECOLI] | 68 | 152 | 2038.8 | 0.0334 | 0.0746 | 2.24 |
| P0A858 | tpiA | Triosephosphate isomerase - Escherichia coli - [TPIS_ECOLI] | 17 | 38 | 2582.1 | 0.0066 | 0.0147 | 2.24 |
| P0A991 | fbaB | Fructose-bisphosphate aldolase class 1 - Escherichia coli - [ALF1_ECOLI] | 39 | 87 | 355.4 | 0.1097 | 0.2448 | 2.23 |
| P21165 | pepQ | Xaa-Pro dipeptidase - Escherichia coli - [PEPQ_ECOLI] | 19 | 42 | 407.5 | 0.0466 | 0.1031 | 2.21 |
| P0AC53 | zwf | Glucose-6-phosphate 1-dehydrogenase - Escherichia coli - [G6PD_ECOLI] | 10 | 22 | 570.7 | 0.0175 | 0.0385 | 2.20 |
| P09372 | grpE | Protein grpE - Escherichia coli - [GRPE_ECOLI] | 10 | 22 | 1431.5 | 0.0070 | 0.0154 | 2.20 |
| P0A9K9 | slyD | FKBP-type peptidyl-prolyl cis-trans isomerase slyD - Escherichia coli - [SLYD_ECOLI] | 5 | 11 | 1783.5 | 0.0028 | 0.0062 | 2.20 |
| P0A698 | uvrA | UvrABC system protein A - Escherichia coli - [UVRA_ECOLI] | 16 | 35 | 35.3 | 0.4538 | 0.9927 | 2.19 |
| P62707 | gpmA | 2.3-bisphosphoglycerate-dependent phosphoglycerate mutase - Escherichia coli - [GPMA_ECOLI] | 38 | 83 | 4968.9 | 0.0076 | 0.0167 | 2.18 |
| P0ACA3 | sspA | Stringent starvation protein A. - Escherichia coli - [SSPA_ECOLI] | 12 | 26 | 648.9 | 0.0185 | 0.0401 | 2.17 |
| P07650 | deoA | Thymidine phosphorylase - Escherichia coli - [TYPH_ECOLI] | 12 | 26 | 63.3 | 0.1895 | 0.4106 | 2.17 |
| P0A6X3 | hfq | Protein hfq - Escherichia coli - [HFQ_ECOLI] | 6 | 13 | 2239.9 | 0.0027 | 0.0058 | 2.17 |
| P00370 | gdhA | NADP-specific glutamate dehydrogenase - Escherichia coli - [DHE4_ECOLI] | 6 | 13 | 1470.1 | 0.0041 | 0.0088 | 2.17 |
| P0ACJ0 | lrp | Leucine-responsive regulatory protein. - Escherichia coli - [LRP_ECOLI] | 6 | 13 | 888.9 | 0.0067 | 0.0146 | 2.17 |
| P22256 | gabT | 4-aminobutyrate aminotransferase - Escherichia coli - [GABT_ECOLI] | 6 | 13 | 67.4 | 0.0890 | 0.1928 | 2.17 |
| P0A9B2 | gapA | Glyceraldehyde-3-phosphate dehydrogenase A - Escherichia coli - [G3P1_ECOLI] | 174 | 368 | 12772.5 | 0.0136 | 0.0288 | 2.11 |
| P0ABP8 | deoD | Purine nucleoside phosphorylase deoD-type - Escherichia coli - [DEOD_ECOLI] | 18 | 38 | 937.5 | 0.0192 | 0.0405 | 2.11 |
| P0A9P0 | lpdA | Dihydrolipoyl dehydrogenase - Escherichia coli - [DLDH_ECOLI] | 28 | 59 | 2275.0 | 0.0123 | 0.0259 | 2.11 |
| P0ABT2 | dps | DNA protection during starvation protein - Escherichia coli - [DPS_ECOLI] | 95 | 200 | 1301.3 | 0.0730 | 0.1537 | 2.11 |
| P63224 | gmhA | Phosphoheptose isomerase - Escherichia coli - [GMHA_ECOLI] | 12 | 25 | 802.8 | 0.0149 | 0.0311 | 2.08 |
| P0A6B7 | iscS | Cysteine desulfurase - Escherichia coli - [ISCS_ECOLI] | 32 | 66 | 1578.0 | 0.0203 | 0.0418 | 2.06 |
| P0A8V2 | rpoB | DNA-directed RNA polymerase subunit beta - Escherichia coli - [RPOB_ECOLI] | 177 | 364 | 2600.8 | 0.0681 | 0.1400 | 2.06 |
| P0A9D8 | dapD | 2.3.4.5-tetrahydropyridine-2.6-dicarboxylate N-succinyltrans - Escherichia coli - [DAPD_ECOLI] | 20 | 41 | 2148.4 | 0.0093 | 0.0191 | 2.05 |
| P09373 | pflB | Formate acetyltransferase 1 - Escherichia coli - [PFLB_ECOLI] | 57 | 115 | 2621.5 | 0.0217 | 0.0439 | 2.02 |
| P0A6F5 | groL | 60 kDa chaperonin - Escherichia coli - [CH60_ECOLI] | 241 | 483 | 10660.6 | 0.0226 | 0.0453 | 2.00 |
| P25516 | acnA | Aconitate hydratase 1 - Escherichia coli - [ACON1_ECOLI] | 86 | 172 | 247.4 | 0.3476 | 0.6953 | 2.00 |
| P33195 | gcvP | Glycine dehydrogenase [decarboxylating] - Escherichia coli - [GCSP_ECOLI] | 45 | 90 | 351.2 | 0.1281 | 0.2562 | 2.00 |
| P0AAI5 | fabF | 3-oxoacyl-[acyl-carrier-protein] synthase 2 - Escherichia coli - [FABF_ECOLI] | 19 | 38 | 381.3 | 0.0498 | 0.0996 | 2.00 |
| P0A6G7 | clpP | ATP-dependent Clp protease proteolytic subunit - Escherichia coli - [CLPP_ECOLI] | 15 | 30 | 1706.9 | 0.0088 | 0.0176 | 2.00 |
| P0AC38 | aspA | Aspartate ammonia-lyase - Escherichia coli - [ASPA_ECOLI] | 11 | 22 | 847.5 | 0.0130 | 0.0260 | 2.00 |
| P35340 | ahpF | Alkyl hydroperoxide reductase subunit F - Escherichia coli - [AHPF_ECOLI] | 10 | 20 | 448.9 | 0.0223 | 0.0446 | 2.00 |
| P0A800 | rpoZ | DNA-directed RNA polymerase subunit omega - Escherichia coli - [RPOZ_ECOLI] | 8 | 16 | 1979.8 | 0.0040 | 0.0081 | 2.00 |
| Q46851 | gpr | Uncharacterized protein yghZ. - Escherichia coli - [YGHZ_ECOLI] | 6 | 12 | 84.8 | 0.0708 | 0.1416 | 2.00 |
| P76187 | ydhF | Oxidoreductase ydhF - Escherichia coli - [YDHF_ECOLI] | 5 | 10 | 31.9 | 0.1566 | 0.3132 | 2.00 |
| P0C0L2 | osmC | Peroxiredoxin osmC - Escherichia coli - [OSMC_ECOLI] | 5 | 10 | 1321.2 | 0.0038 | 0.0076 | 2.00 |
| P0AE08 | ahpC | Alkyl hydroperoxide reductase subunit C - Escherichia coli - [AHPC_ECOLI] | 36 | 71 | 4408.1 | 0.0082 | 0.0161 | 1.97 |
| P25553 | aldA | Aldehyde dehydrogenase A - Escherichia coli - [ALDA_ECOLI] | 69 | 134 | 606.8 | 0.1137 | 0.2208 | 1.94 |
| P00959 | metG | Methionyl-tRNA synthetase - Escherichia coli - [SYM_ECOLI] | 25 | 48 | 462.4 | 0.0541 | 0.1038 | 1.92 |
| P04805 | gltX | Glutamyl-tRNA synthetase - Escherichia coli - [SYE_ECOLI] | 23 | 44 | 735.4 | 0.0313 | 0.0598 | 1.91 |
| P27298 | prlC | Oligopeptidase A - Escherichia coli - [OPDA_ECOLI] | 11 | 21 | 311.9 | 0.0353 | 0.0673 | 1.91 |
| P0A6L2 | dapA | Dihydrodipicolinate synthase - Escherichia coli - [DAPA_ECOLI] | 11 | 21 | 570.5 | 0.0193 | 0.0368 | 1.91 |
| P0A9Q5 | accD | Acetyl-coenzyme A carboxylase carboxyl transferase subunit b - Escherichia coli - [ACCD_ECOLI] | 10 | 19 | 794.9 | 0.0126 | 0.0239 | 1.90 |
| P08312 | pheS | Phenylalanyl-tRNA synthetase alpha chain - Escherichia coli - [SYFA_ECOLI] | 9 | 17 | 635.6 | 0.0142 | 0.0267 | 1.89 |
| P0A867 | talA | Transaldolase A - Escherichia coli - [TALA_ECOLI] | 17 | 32 | 520.1 | 0.0327 | 0.0615 | 1.88 |
| P0ABK5 | cysK | Cysteine synthase A - Escherichia coli - [CYSK_ECOLI] | 24 | 45 | 4105.8 | 0.0058 | 0.0110 | 1.88 |
| P0A8G6 | wrbA | Flavoprotein wrbA - Escherichia coli - [WRBA_ECOLI] | 22 | 41 | 1082.3 | 0.0203 | 0.0379 | 1.86 |
| P05055 | pnp | Polyribonucleotide nucleotidyltransferase - Escherichia coli - [PNP_ECOLI] | 102 | 190 | 1653.4 | 0.0617 | 0.1149 | 1.86 |
| P0AFP6 | ybgI | UPF0135 protein ybgI. - Escherichia coli - [YBGI_ECOLI] | 7 | 13 | 349.6 | 0.0200 | 0.0372 | 1.86 |
| P0ABD3 | bfr | Bacterioferritin - Escherichia coli - [BFR_ECOLI] | 30 | 55 | 333.1 | 0.0901 | 0.1651 | 1.83 |
| P0AAI9 | fabD | Malonyl CoA-acyl carrier protein transacylase - Escherichia coli - [FABD_ECOLI] | 6 | 11 | 1088.0 | 0.0055 | 0.0101 | 1.83 |
| P0A805 | frr | Ribosome recycling factor - Escherichia coli - [RRF_ECOLI] | 11 | 20 | 1764.7 | 0.0062 | 0.0113 | 1.82 |
| P27302 | tktA | Transketolase 1 - Escherichia coli - [TKT1_ECOLI] | 38 | 69 | 1151.4 | 0.0330 | 0.0599 | 1.82 |
| P0A6A3 | ackA | Acetate kinase - Escherichia coli - [ACKA_ECOLI] | 31 | 56 | 861.4 | 0.0360 | 0.0650 | 1.81 |
| P0AC41 | sdhA | Succinate dehydrogenase flavoprotein subunit - Escherichia coli - [DHSA_ECOLI] | 46 | 83 | 394.3 | 0.1167 | 0.2105 | 1.80 |
| P25526 | gabD | Succinate-semialdehyde dehydrogenase [NADP+] - Escherichia coli - [GABD_ECOLI] | 10 | 18 | 102.8 | 0.0973 | 0.1751 | 1.80 |
| P0A6T1 | pgi | Glucose-6-phosphate isomerase - Escherichia coli - [G6PI_ECOLI] | 10 | 18 | 1279.5 | 0.0078 | 0.0141 | 1.80 |
| P33599 | nuoC | NADH-quinone oxidoreductase subunit C/D - Escherichia coli - [NUOCD_ECOLI] | 14 | 25 | 118.0 | 0.1186 | 0.2119 | 1.79 |
| P37330 | glcB | Malate synthase G - Escherichia coli - [MASZ_ECOLI] | 23 | 41 | 135.0 | 0.1703 | 0.3036 | 1.78 |
| P0AET8 | hdhA | 7-alpha-hydroxysteroid dehydrogenase - Escherichia coli - [HDHA_ECOLI] | 9 | 16 | 135.3 | 0.0665 | 0.1183 | 1.78 |
| P07395 | pheT | Phenylalanyl-tRNA synthetase beta chain - Escherichia coli - [SYFB_ECOLI] | 38 | 67 | 486.1 | 0.0782 | 0.1378 | 1.76 |
| P0AF93 | ridA | UPF0076 protein yjgF. - Escherichia coli - [YJGF_ECOLI] | 8 | 14 | 3910.7 | 0.0020 | 0.0036 | 1.75 |
| P26616 | maeA | NAD-dependent malic enzyme - Escherichia coli - [MAO1_ECOLI] | 26 | 45 | 168.4 | 0.1544 | 0.2672 | 1.73 |
| P0ABH7 | gltA | Citrate synthase - Escherichia coli - [CISY_ECOLI] | 61 | 104 | 1232.3 | 0.0495 | 0.0844 | 1.70 |
| P0A6P1 | tsf | Elongation factor Ts - Escherichia coli - [EFTS_ECOLI] | 50 | 85 | 4006.8 | 0.0125 | 0.0212 | 1.70 |
| P0A717 | prs | Ribose-phosphate pyrophosphokinase - Escherichia coli - [KPRS_ECOLI] | 30 | 51 | 1511.2 | 0.0199 | 0.0337 | 1.70 |
| P00962 | glnS | Glutaminyl-tRNA synthetase - Escherichia coli - [SYQ_ECOLI] | 10 | 17 | 511.2 | 0.0196 | 0.0333 | 1.70 |
| P00957 | alaS | Alanyl-tRNA synthetase - Escherichia coli - [SYA_ECOLI] | 49 | 83 | 626.1 | 0.0783 | 0.1326 | 1.69 |
| P0ADG7 | guaB | Inosine-5'-monophosphate dehydrogenase - Escherichia coli - [IMDH_ECOLI] | 19 | 32 | 1721.1 | 0.0110 | 0.0186 | 1.68 |
| Q46857 | dkgA | 2.5-diketo-D-gluconic acid reductase A - Escherichia coli - [DKGA_ECOLI] | 12 | 20 | 250.0 | 0.0480 | 0.0800 | 1.67 |
| P24171 | dcp | Peptidyl-dipeptidase dcp - Escherichia coli - [DCP_ECOLI] | 6 | 10 | 173.4 | 0.0346 | 0.0577 | 1.67 |
| P33643 | rluD | Ribosomal large subunit pseudouridine synthase D - Escherichia coli - [RLUD_ECOLI] | 6 | 10 | 45.4 | 0.1321 | 0.2201 | 1.67 |
| P52697 | pgl | 6-phosphogluconolactonase - Escherichia coli - [6PGL_ECOLI] | 6 | 10 | 539.7 | 0.0111 | 0.0185 | 1.67 |
| P07004 | proA | Gamma-glutamyl phosphate reductase - Escherichia coli - [PROA_ECOLI] | 6 | 10 | 146.3 | 0.0410 | 0.0683 | 1.67 |
| P0AGE0 | ssb | Single-stranded DNA-binding protein - Escherichia coli - [SSB_ECOLI] | 6 | 10 | 278.7 | 0.0215 | 0.0359 | 1.67 |
| P0A6Z3 | htpG | Chaperone protein htpG - Escherichia coli - [HTPG_ECOLI] | 99 | 164 | 701.3 | 0.1412 | 0.2339 | 1.66 |
| P0AFG8 | aceE | Pyruvate dehydrogenase E1 component - Escherichia coli - [ODP1_ECOLI] | 90 | 149 | 2084.7 | 0.0432 | 0.0715 | 1.66 |
| P39177 | uspG | Universal stress protein G. - Escherichia coli - [USPG_ECOLI] | 23 | 38 | 1418.9 | 0.0162 | 0.0268 | 1.65 |
| P21889 | aspS | Aspartyl-tRNA synthetase - Escherichia coli - [SYD_ECOLI] | 30 | 49 | 863.3 | 0.0348 | 0.0568 | 1.63 |
| P21499 | rnr | Ribonuclease R - Escherichia coli - [RNR_ECOLI] | 19 | 31 | 230.7 | 0.0824 | 0.1344 | 1.63 |
| P0A853 | tnaA | Tryptophanase - Escherichia coli - [TNAA_ECOLI] | 125 | 202 | 2619.7 | 0.0477 | 0.0771 | 1.62 |
| P0A799 | pgk | Phosphoglycerate kinase - Escherichia coli - [PGK_ECOLI] | 128 | 205 | 8342.7 | 0.0153 | 0.0246 | 1.60 |
| P08200 | icd | Isocitrate dehydrogenase [NADP] - Escherichia coli - [IDH_ECOLI] | 65 | 104 | 4673.8 | 0.0139 | 0.0223 | 1.60 |
| P0AEZ9 | moaB | Molybdenum cofactor biosynthesis protein B. - Escherichia coli - [MOAB_ECOLI] | 10 | 16 | 259.7 | 0.0385 | 0.0616 | 1.60 |
| P0A6R0 | fabH | 3-oxoacyl-[acyl-carrier-protein] synthase 3 - Escherichia coli - [FABH_ECOLI] | 10 | 16 | 821.3 | 0.0122 | 0.0195 | 1.60 |
| P00363 | frdA | Fumarate reductase flavoprotein subunit - Escherichia coli - [FRDA_ECOLI] | 5 | 8 | 99.7 | 0.0501 | 0.0802 | 1.60 |
| P0ACY1 | ydjA | Protein ydjA. - Escherichia coli - [YDJA_ECOLI] | 5 | 8 | 539.5 | 0.0093 | 0.0148 | 1.60 |
| P0AE01 | trmJ | Uncharacterized tRNA/rRNA methyltransferase yfhQ - Escherichia coli - [YFHQ_ECOLI] | 5 | 8 | 195.5 | 0.0256 | 0.0409 | 1.60 |
| P60651 | speB | Agmatinase - Escherichia coli - [SPEB_ECOLI] | 5 | 8 | 296.1 | 0.0169 | 0.0270 | 1.60 |
| P77395 | ybbN | Uncharacterized protein ybbN. - Escherichia coli - [YBBN_ECOLI] | 5 | 8 | 537.4 | 0.0093 | 0.0149 | 1.60 |
| P0A8M0 | asnS | Asparaginyl-tRNA synthetase - Escherichia coli - [SYN_ECOLI] | 49 | 78 | 1232.7 | 0.0398 | 0.0633 | 1.59 |
| P0ABD5 | accA | Acetyl-coenzyme A carboxylase carboxyl transferase subunit a - Escherichia coli - [ACCA_ECOLI] | 22 | 35 | 944.0 | 0.0233 | 0.0371 | 1.59 |
| P0ABA6 | atpG | ATP synthase gamma chain - Escherichia coli - [ATPG_ECOLI] | 7 | 11 | 762.7 | 0.0092 | 0.0144 | 1.57 |
| P69924 | nrdB | Ribonucleoside-diphosphate reductase 1 subunit beta - Escherichia coli - [RIR2_ECOLI] | 7 | 11 | 158.2 | 0.0442 | 0.0695 | 1.57 |
| P07118 | valS | Valyl-tRNA synthetase - Escherichia coli - [SYV_ECOLI] | 39 | 61 | 455.2 | 0.0857 | 0.1340 | 1.56 |
| P0ADG4 | suhB | Inositol-1-monophosphatase - Escherichia coli - [SUHB_ECOLI] | 9 | 14 | 382.3 | 0.0235 | 0.0366 | 1.56 |
| P68767 | pepA | Cytosol aminopeptidase - Escherichia coli - [AMPA_ECOLI] | 11 | 17 | 286.4 | 0.0384 | 0.0593 | 1.55 |
| P67910 | hldD | ADP-L-glycero-D-manno-heptose-6-epimerase - Escherichia coli - [HLDD_ECOLI] | 15 | 23 | 685.7 | 0.0219 | 0.0335 | 1.53 |
| P0AEK4 | fabI | Enoyl-[acyl-carrier-protein] reductase [NADH] - Escherichia coli - [FABI_ECOLI] | 25 | 38 | 1679.8 | 0.0149 | 0.0226 | 1.52 |
| P0AG67 | rpsA | 30S ribosomal protein S1. - Escherichia coli - [RS1_ECOLI] | 124 | 188 | 2649.1 | 0.0468 | 0.0710 | 1.52 |
| P0A7Z4 | rpoA | DNA-directed RNA polymerase subunit alpha - Escherichia coli - [RPOA_ECOLI] | 72 | 109 | 2981.5 | 0.0241 | 0.0366 | 1.51 |
| P22259 | pckA | Phosphoenolpyruvate carboxykinase [ATP] - Escherichia coli - [PPCK_ECOLI] | 20 | 30 | 628.3 | 0.0318 | 0.0477 | 1.50 |
| P0A9A9 | fur | Ferric uptake regulation protein - Escherichia coli - [FUR_ECOLI] | 12 | 18 | 575.4 | 0.0209 | 0.0313 | 1.50 |
| P23721 | serC | Phosphoserine aminotransferase - Escherichia coli - [SERC_ECOLI] | 8 | 12 | 1716.2 | 0.0047 | 0.0070 | 1.50 |
| P0AE88 | cpxR | Transcriptional regulatory protein cpxR. - Escherichia coli - [CPXR_ECOLI] | 6 | 9 | 566.3 | 0.0106 | 0.0159 | 1.50 |
| P06999 | pfkB | 6-phosphofructokinase isozyme 2 - Escherichia coli - [K6PF2_ECOLI] | 6 | 9 | 147.7 | 0.0406 | 0.0610 | 1.50 |
| P0A6Y8 | dnaK | Chaperone protein dnaK - Escherichia coli - [DNAK_ECOLI] | 247 | 370 | 8986.8 | 0.0275 | 0.0412 | 1.50 |
| P0A7D4 | purA | Adenylosuccinate synthetase - Escherichia coli - [PURA_ECOLI] | 35 | 52 | 2503.4 | 0.0140 | 0.0208 | 1.49 |
| P0ABD8 | accB | Biotin carboxyl carrier protein of acetyl-CoA carboxylase - Escherichia coli - [BCCP_ECOLI] | 42 | 62 | 657.1 | 0.0639 | 0.0943 | 1.48 |
| P0AD61 | pykF | Pyruvate kinase I - Escherichia coli - [KPYK1_ECOLI] | 42 | 62 | 1228.6 | 0.0342 | 0.0505 | 1.48 |
| P0A6H5 | hslU | ATP-dependent hsl protease ATP-binding subunit hslU - Escherichia coli - [HSLU_ECOLI] | 22 | 32 | 483.0 | 0.0455 | 0.0662 | 1.45 |
| P0A9Q9 | asd | Aspartate-semialdehyde dehydrogenase - Escherichia coli - [DHAS_ECOLI] | 11 | 16 | 1792.0 | 0.0061 | 0.0089 | 1.45 |
| P69783 | crr | Glucose-specific phosphotransferase enzyme IIA component - Escherichia coli - [PTGA_ECOLI] | 19 | 27 | 4167.9 | 0.0046 | 0.0065 | 1.42 |
| P0AES4 | gyrA | DNA gyrase subunit A - Escherichia coli - [GYRA_ECOLI] | 49 | 69 | 412.7 | 0.1187 | 0.1672 | 1.41 |
| P0ACF8 | hns | DNA-binding protein H-NS - Escherichia coli - [HNS_ECOLI] | 35 | 49 | 7477.0 | 0.0047 | 0.0066 | 1.40 |
| P0A870 | talB | Transaldolase B - Escherichia coli - [TALB_ECOLI] | 30 | 42 | 2457.4 | 0.0122 | 0.0171 | 1.40 |
| P61889 | mdh | Malate dehydrogenase - Escherichia coli - [MDH_ECOLI] | 30 | 42 |  |  |  | 1.40 |
| P29012 | dadX | Alanine racemase. catabolic - Escherichia coli - [ALR2_ECOLI] | 5 | 7 | 21.4 | 0.2333 | 0.3267 | 1.40 |
| P11875 | argS | Arginyl-tRNA synthetase - Escherichia coli - [SYR_ECOLI] | 5 | 7 | 97.4 | 0.0513 | 0.0718 | 1.40 |
| P37744 | rmlA1 | Glucose-1-phosphate thymidylyltransferase 1 - Escherichia coli - [RMLA1_ECOLI] | 8 | 11 |  |  |  | 1.38 |
| P0A7K2 | rplL | 50S ribosomal protein L7/L12 - Escherichia coli - [RL7_ECOLI] | 48 | 65 | 14543.5 | 0.0033 | 0.0045 | 1.35 |
| P0A6P9 | eno | Enolase - Escherichia coli - [ENO_ECOLI] | 56 | 75 | 11218.0 | 0.0050 | 0.0067 | 1.34 |
| P69797 | manX | PTS system mannose-specific EIIAB component - Escherichia coli - [PTNAB_ECOLI] | 15 | 20 | 804.8 | 0.0186 | 0.0249 | 1.33 |
| P29745 | pepT | Peptidase T - Escherichia coli - [PEPT_ECOLI] | 6 | 8 | 129.0 | 0.0465 | 0.0620 | 1.33 |
| P60906 | hisS | Histidyl-tRNA synthetase - Escherichia coli - [SYH_ECOLI] | 23 | 30 | 432.7 | 0.0532 | 0.0693 | 1.30 |
| P0A825 | glyA | Serine hydroxymethyltransferase - Escherichia coli - [GLYA_ECOLI] | 10 | 13 | 7872.7 | 0.0013 | 0.0017 | 1.30 |
| P04079 | guaA | GMP synthase [glutamine-hydrolyzing] - Escherichia coli - [GUAA_ECOLI] | 31 | 40 | 1224.3 | 0.0253 | 0.0327 | 1.29 |
| P0AGE9 | sucD | Succinyl-CoA ligase [ADP-forming] subunit alpha - Escherichia coli - [SUCD_ECOLI] | 7 | 9 | 1950.1 | 0.0036 | 0.0046 | 1.29 |
| P69441 | adk | Adenylate kinase - Escherichia coli - [KAD_ECOLI] | 7 | 9 | 4676.0 | 0.0015 | 0.0019 | 1.29 |
| P76268 | kdgR | Transcriptional regulator kdgR. - Escherichia coli - [KDGR_ECOLI] | 7 | 9 | 63.6 | 0.1101 | 0.1416 | 1.29 |
| P37192 | gatY | Tagatose-1.6-bisphosphate aldolase gatY - Escherichia coli - [GATY_ECOLI] | 36 | 45 |  |  |  | 1.25 |
| P0A9L3 | fklB | FKBP-type 22 kDa peptidyl-prolyl cis-trans isomerase - Escherichia coli - [FKBB_ECOLI] | 12 | 15 | 621.3 | 0.0193 | 0.0241 | 1.25 |
| P00509 | aspC | Aspartate aminotransferase - Escherichia coli - [AAT_ECOLI] | 8 | 10 | 1643.4 | 0.0049 | 0.0061 | 1.25 |
| P0ABF6 | cdd | Cytidine deaminase - Escherichia coli - [CDD_ECOLI] | 8 | 10 | 96.1 | 0.0833 | 0.1041 | 1.25 |
| P04951 | kdsB | 3-deoxy-manno-octulosonate cytidylyltransferase - Escherichia coli - [KDSB_ECOLI] | 8 | 10 | 97.2 | 0.0823 | 0.1029 | 1.25 |
| P0A7J3 | rplJ | 50S ribosomal protein L10 - Escherichia coli - [RL10_ECOLI] | 34 | 42 | 7731.6 | 0.0044 | 0.0054 | 1.24 |
| P27248 | gcvT | Aminomethyltransferase - Escherichia coli - [GCST_ECOLI] | 13 | 16 | 428.5 | 0.0303 | 0.0373 | 1.23 |
| P15288 | pepD | Aminoacyl-histidine dipeptidase - Escherichia coli - [PEPD_ECOLI] | 14 | 17 | 1015.8 | 0.0138 | 0.0167 | 1.21 |
| P00579 | rpoD | RNA polymerase sigma factor rpoD - Escherichia coli - [RPOD_ECOLI] | 15 | 18 | 310.2 | 0.0484 | 0.0580 | 1.20 |
| P00448 | sodA | Superoxide dismutase [Mn] - Escherichia coli - [SODM_ECOLI] | 6 | 7 | 1594.0 | 0.0038 | 0.0044 | 1.17 |
| P0AE12 | amn | AMP nucleosidase - Escherichia coli - [AMN_ECOLI] | 6 | 7 | 39.6 | 0.1517 | 0.1769 | 1.17 |
| P0A817 | metK | S-adenosylmethionine synthetase - Escherichia coli - [METK_ECOLI] | 14 | 16 | 2620.1 | 0.0053 | 0.0061 | 1.14 |
| P76113 | curA | Putative NADP-dependent oxidoreductase yncB - Escherichia coli - [YNCB_ECOLI] | 7 | 8 | 66.5 | 0.1052 | 0.1202 | 1.14 |
| P0A707 | infC | Translation initiation factor IF-3. - Escherichia coli - [IF3_ECOLI] | 43 | 49 | 2222.5 | 0.0193 | 0.0220 | 1.14 |
| P45578 | luxS | S-ribosylhomocysteine lyase - Escherichia coli - [LUXS_ECOLI] | 8 | 9 | 1624.8 | 0.0049 | 0.0055 | 1.13 |
| P0A6K6 | deoB | Phosphopentomutase - Escherichia coli - [DEOB_ECOLI] | 25 | 28 | 872.3 | 0.0287 | 0.0321 | 1.12 |
| P69503 | apt | Adenine phosphoribosyltransferase - Escherichia coli - [APT_ECOLI] | 9 | 10 | 363.0 | 0.0248 | 0.0275 | 1.11 |
| P05804 | uidA | Beta-glucuronidase - Escherichia coli - [BGLR_ECOLI] | 9 | 10 | 1.8 | 4.9799 | 5.5333 | 1.11 |
| P61714 | ribE | 6.7-dimethyl-8-ribityllumazine synthase - Escherichia coli - [RISB_ECOLI] | 10 | 11 | 1955.3 | 0.0051 | 0.0056 | 1.10 |
| P0A953 | fabB | 3-oxoacyl-[acyl-carrier-protein] synthase 1 - Escherichia coli - [FABB_ECOLI] | 85 | 92 | 1776.8 | 0.0478 | 0.0518 | 1.08 |
| P0A7W1 | rpsE | 30S ribosomal protein S5. - Escherichia coli - [RS5_ECOLI] | 27 | 28 | 7657.3 | 0.0035 | 0.0037 | 1.04 |
| P0A7R1 | rplI | 50S ribosomal protein L9. - Escherichia coli - [RL9_ECOLI] | 20 | 20 | 7434.8 | 0.0027 | 0.0027 | 1.00 |
| P0A8E7 | yajQ | UPF0234 protein yajQ. - Escherichia coli - [YAJQ_ECOLI] | 14 | 14 | 1209.5 | 0.0116 | 0.0116 | 1.00 |
| P09148 | galT | Galactose-1-phosphate uridylyltransferase - Escherichia coli - [GAL7_ECOLI] | 12 | 12 | 1912.2 | 0.0063 | 0.0063 | 1.00 |
| P0AFG0 | nusG | Transcription antitermination protein nusG. - Escherichia coli - [NUSG_ECOLI] | 12 | 12 | 682.1 | 0.0176 | 0.0176 | 1.00 |
| P68066 | grcA | Autonomous glycyl radical cofactor. - Escherichia coli - [GRCA_ECOLI] | 11 | 11 | 4400.9 | 0.0025 | 0.0025 | 1.00 |
| P0A862 | tpx | Thiol peroxidase - Escherichia coli - [TPX_ECOLI] | 11 | 11 | 4678.2 | 0.0024 | 0.0024 | 1.00 |
| P0A7T3 | rpsP | 30S ribosomal protein S16. - Escherichia coli - [RS16_ECOLI] | 10 | 10 | 6611.2 | 0.0015 | 0.0015 | 1.00 |
| P09158 | speE | Spermidine synthase - Escherichia coli - [SPEE_ECOLI] | 7 | 7 | 181.6 | 0.0385 | 0.0385 | 1.00 |
| P10121 | ftsY | Cell division protein ftsY. - Escherichia coli - [FTSY_ECOLI] | 7 | 7 | 190.5 | 0.0367 | 0.0367 | 1.00 |
| P07913 | tdh | L-threonine 3-dehydrogenase - Escherichia coli - [TDH_ECOLI] | 7 | 7 | 133.6 | 0.0524 | 0.0524 | 1.00 |
| P0A6W9 | gshA | Glutamate--cysteine ligase - Escherichia coli - [GSH1_ECOLI] | 7 | 7 | 131.7 | 0.0532 | 0.0532 | 1.00 |
| P30850 | rnb | Exoribonuclease 2 - Escherichia coli - [RNB_ECOLI] | 6 | 6 | 231.1 | 0.0260 | 0.0260 | 1.00 |
| P0A9S1 | fucO | Lactaldehyde reductase - Escherichia coli - [FUCO_ECOLI] | 5 | 5 | 25.1 | 0.1989 | 0.1989 | 1.00 |
| P0A6W5 | greA | Transcription elongation factor greA - Escherichia coli - [GREA_ECOLI] | 5 | 5 | 496.0 | 0.0101 | 0.0101 | 1.00 |
| P37647 | kdgK | 2-dehydro-3-deoxygluconokinase - Escherichia coli - [KDGK_ECOLI] | 5 | 5 | 50.3 | 0.0995 | 0.0995 | 1.00 |
| P17445 | betB | Betaine aldehyde dehydrogenase - Escherichia coli - [BETB_ECOLI] | 5 | 5 | 23.8 | 0.2099 | 0.2099 | 1.00 |
| P0A9D2 | gstA | Glutathione S-transferase - Escherichia coli - [GST_ECOLI] | 5 | 5 | 238.6 | 0.0210 | 0.0210 | 1.00 |
| P24182 | accC | Biotin carboxylase - Escherichia coli - [ACCC_ECOLI] | 25 | 24 | 397.8 | 0.0629 | 0.0603 | 0.96 |
| P0AB71 | fbaA | Fructose-bisphosphate aldolase class 2 - Escherichia coli - [ALF_ECOLI] | 21 | 20 | 2870.0 | 0.0073 | 0.0070 | 0.95 |
| P04825 | pepN | Aminopeptidase N - Escherichia coli - [AMPN_ECOLI] | 45 | 42 | 365.4 | 0.1231 | 0.1149 | 0.93 |
| P17169 | glmS | Glucosamine--fructose-6-phosphate aminotransferase [isomeriz - Escherichia coli - [GLMS_ECOLI] | 20 | 18 | 340.6 | 0.0587 | 0.0528 | 0.90 |
| P0AED0 | uspA | Universal stress protein A. - Escherichia coli - [USPA_ECOLI] | 10 | 9 | 1511.1 | 0.0066 | 0.0060 | 0.90 |
| P08997 | aceB | Malate synthase A - Escherichia coli - [MASY_ECOLI] | 125 | 112 | 810.0 | 0.1543 | 0.1383 | 0.90 |
| P0ABA0 | atpF | ATP synthase B chain - Escherichia coli - [ATPF_ECOLI] | 8 | 7 | 1009.8 | 0.0079 | 0.0069 | 0.88 |
| P36683 | acnB | Aconitate hydratase 2 - Escherichia coli - [ACON2_ECOLI] | 77 | 67 | 1251.9 | 0.0615 | 0.0535 | 0.87 |
| P0A6Q3 | fabA | 3-hydroxydecanoyl-[acyl-carrier-protein] dehydratase - Escherichia coli - [FABA_ECOLI] | 14 | 12 | 1261.2 | 0.0111 | 0.0095 | 0.86 |
| P62620 | ispG | 4-hydroxy-3-methylbut-2-en-1-yl diphosphate synthase - Escherichia coli - [ISPG_ECOLI] | 7 | 6 | 407.7 | 0.0172 | 0.0147 | 0.86 |
| P0A7G2 | rbfA | Ribosome-binding factor A - Escherichia coli - [RBFA_ECOLI] | 7 | 6 | 583.0 | 0.0120 | 0.0103 | 0.86 |
| P37095 | pepB | Peptidase B - Escherichia coli - [PEPB_ECOLI] | 19 | 16 | 399.5 | 0.0476 | 0.0400 | 0.84 |
| P0A8W8 | yfbU | UPF0304 protein yfbU. - Escherichia coli - [YFBU_ECOLI] | 12 | 10 | 454.2 | 0.0264 | 0.0220 | 0.83 |
| P0A9M5 | gpt | Xanthine phosphoribosyltransferase - Escherichia coli - [XGPT_ECOLI] | 6 | 5 | 105.3 | 0.0570 | 0.0475 | 0.83 |
| P68919 | rplY | 50S ribosomal protein L25. - Escherichia coli - [RL25_ECOLI] | 6 | 5 | 4246.4 | 0.0014 | 0.0012 | 0.83 |
| P31658 | hchA | Chaperone protein hchA - Escherichia coli - [HCHA_ECOLI] | 16 | 13 | 404.1 | 0.0396 | 0.0322 | 0.81 |
| P0A8F0 | upp | Uracil phosphoribosyltransferase - Escherichia coli - [UPP_ECOLI] | 14 | 11 | 2298.8 | 0.0061 | 0.0048 | 0.79 |
| P36938 | pgm | Phosphoglucomutase - Escherichia coli - [PGM_ECOLI] | 9 | 7 | 409.6 | 0.0220 | 0.0171 | 0.78 |
| P0AA10 | rplM | 50S ribosomal protein L13. - Escherichia coli - [RL13_ECOLI] | 9 | 7 | 4268.0 | 0.0021 | 0.0016 | 0.78 |
| P0AEK2 | fabG | 3-oxoacyl-[acyl-carrier-protein] reductase - Escherichia coli - [FABG_ECOLI] | 21 | 16 | 828.3 | 0.0254 | 0.0193 | 0.76 |
| P02358 | rpsF | 30S ribosomal protein S6 - Escherichia coli - [RS6_ECOLI] | 12 | 9 | 4732.1 | 0.0025 | 0.0019 | 0.75 |
| P0AD12 | yeeZ | Protein yeeZ - Escherichia coli - [YEEZ_ECOLI] | 8 | 6 | 112.7 | 0.0710 | 0.0532 | 0.75 |
| P0A9T0 | serA | D-3-phosphoglycerate dehydrogenase - Escherichia coli - [SERA_ECOLI] | 8 | 6 | 2584.3 | 0.0031 | 0.0023 | 0.75 |
| P37689 | gpmI | 2.3-bisphosphoglycerate-independent phosphoglycerate mutase - Escherichia coli - [GPMI_ECOLI] | 15 | 11 | 627.2 | 0.0239 | 0.0175 | 0.73 |
| P0AE18 | map | Methionine aminopeptidase - Escherichia coli - [AMPM_ECOLI] | 7 | 5 | 388.2 | 0.0180 | 0.0129 | 0.71 |
| P37765 | rluB | Ribosomal large subunit pseudouridine synthase B - Escherichia coli - [RLUB_ECOLI] | 7 | 5 | 98.5 | 0.0711 | 0.0508 | 0.71 |
| P0A7E5 | pyrG | CTP synthase - Escherichia coli - [PYRG_ECOLI] | 7 | 5 | 638.6 | 0.0110 | 0.0078 | 0.71 |
| P0A9K3 | ybeZ | PhoH-like protein. - Escherichia coli - [PHOL_ECOLI] | 17 | 11 | 226.4 | 0.0751 | 0.0486 | 0.65 |
| P0A850 | tig | Trigger factor - Escherichia coli - [TIG_ECOLI] | 158 | 102 | 3826.4 | 0.0413 | 0.0267 | 0.65 |
| P0AFF6 | nusA | Transcription elongation protein nusA - Escherichia coli - [NUSA_ECOLI] | 55 | 33 | 1125.1 | 0.0489 | 0.0293 | 0.60 |
| P16659 | proS | Prolyl-tRNA synthetase - Escherichia coli - [SYP_ECOLI] | 27 | 16 | 707.8 | 0.0381 | 0.0226 | 0.59 |
| P0A836 | sucC | Succinyl-CoA synthetase beta chain - Escherichia coli - [SUCC_ECOLI] | 23 | 12 | 2211.5 | 0.0104 | 0.0054 | 0.52 |
| P0AGJ9 | tyrS | Tyrosyl-tRNA synthetase - Escherichia coli - [SYY_ECOLI] | 12 | 6 | 534.7 | 0.0224 | 0.0112 | 0.50 |
| P0AG44 | rplQ | 50S ribosomal protein L17. - Escherichia coli - [RL17_ECOLI] | 28 | 13 | 4272.8 | 0.0066 | 0.0030 | 0.46 |
| P0A7L0 | rplA | 50S ribosomal protein L1. - Escherichia coli - [RL1_ECOLI] | 38 | 15 | 3867.2 | 0.0098 | 0.0039 | 0.39 |
| P0A7R5 | rpsJ | 30S ribosomal protein S10. - Escherichia coli - [RS10_ECOLI] | 18 | 7 | 3472.7 | 0.0052 | 0.0020 | 0.39 |
| P0C054 | ibpA | Small heat shock protein ibpA - Escherichia coli - [IBPA_ECOLI] | 13 | 5 | 299.0 | 0.0435 | 0.0167 | 0.38 |
| P60438 | rplC | 50S ribosomal protein L3. - Escherichia coli - [RL3_ECOLI] | 24 | 9 | 4460.3 | 0.0054 | 0.0020 | 0.38 |
| P23836 | phoP | Transcriptional regulatory protein phoP. - Escherichia coli - [PHOP_ECOLI] | 19 | 7 | 449.1 | 0.0423 | 0.0156 | 0.37 |
| P0A7R9 | rpsK | 30S ribosomal protein S11. - Escherichia coli - [RS11_ECOLI] | 14 | 5 | 2900.5 | 0.0048 | 0.0017 | 0.36 |
| P0A7V3 | rpsC | 30S ribosomal protein S3. - Escherichia coli - [RS3_ECOLI] | 20 | 5 | 5755.0 | 0.0035 | 0.0009 | 0.25 |
| P60422 | rplB | 50S ribosomal protein L2. - Escherichia coli - [RL2_ECOLI] | 30 | 7 | 5658.4 | 0.0053 | 0.0012 | 0.23 |
| P02359 | rpsG | 30S ribosomal protein S7. - Escherichia coli - [RS7_ECOLI] | 67 | 15 | 8660.2 | 0.0077 | 0.0017 | 0.22 |
| P0A9M8 | pta | Phosphate acetyltransferase - Escherichia coli - [PTA_ECOLI] | 37 | 8 | 581.3 | 0.0637 | 0.0138 | 0.22 |
| P0A9G6 | aceA | Isocitrate lyase - Escherichia coli - [ACEA_ECOLI] | 156 | 29 | 3745.5 | 0.0416 | 0.0077 | 0.19 |
| P0A7V8 | rpsD | 30S ribosomal protein S4. - Escherichia coli - [RS4_ECOLI] | 49 | 7 | 5187.4 | 0.0094 | 0.0013 | 0.14 |
| P0A7S9 | rpsM | 30S ribosomal protein S13. - Escherichia coli - [RS13_ECOLI] | 35 | 5 | 5733.1 | 0.0061 | 0.0009 | 0.14 |

**Table S4.**

|  | **UniProt AC** | **Gene name** | **Protein name** | **Factor of change (S/NS)** | **pI** |
| --- | --- | --- | --- | --- | --- |
| **Increased Interaction potential (IIP)** | P33570 | tktB | Transketolase 2  Escherichia coli - [TKT2_ECOLI] | 5,3 | **5.86** |
|  | P09152 | narG | Respiratory nitrate reductase 1 alpha chain  Escherichia coli - [NARG_ECOLI] | 4,3 | **6.05** |
|  | P21599 | pykA | Pyruvate kinase II  Escherichia coli - [KPYK2_ECOLI] | 4,2 | **6.23** |
|  | P07014 | sdhB | Succinate dehydrogenase iron-sulfur subunit  Escherichia coli - [DHSB_ECOLI] | 3,8 | **6.31** |
|  | P0A6L0 | deoC | Deoxyribose-phosphate aldolase  Escherichia coli - [DEOC_ECOLI] | 3,8 | **5.5** |
|  | P12758 | udp | Uridine phosphorylase  Escherichia coli - [UDP_ECOLI] | 3,6 | **5.81** |
|  | P32176 | fdoG | Formate dehydrogenase-O. major subunit  Escherichia coli - [FDOG_ECOLI] | 3,6 | **6.54** |
|  | P0A8L1 | serS | Seryl-tRNA synthetase  Escherichia coli - [SYS_ECOLI] | 3,4 | **5.34** |
|  | P23839 | yicC | Protein yicC.  Escherichia coli - [YICC_ECOLI] | 3,2 | **5.08** |
|  | P0ABB4 | atpD | ATP synthase subunit beta  Escherichia coli - [ATPB_ECOLI] | 3,1 | **4.9** |
|  | P33602 | nuoG | NADH-quinone oxidoreductase subunit G Escherichia coli - [NUOG_ECOLI] | 3,0 | **5.85** |
|  | P61517 | can | Carbonic anhydrase 2  Escherichia coli - [CAN_ECOLI] | 3,0 | **6.16** |
|  | P76558 | maeB | NADP-dependent malic enzyme  Escherichia coli - [MAO2_ECOLI] | 2,9 | **5.34** |
|  | P06959 | aceF | Dihydrolipoyllysine-residue acetyltransferase component of p  Escherichia coli - [ODP2_ECOLI] | 2,9 | **5.09** |
|  | P0A9C5 | glnA | Glutamine synthetase  Escherichia coli - [GLNA_ECOLI] | 2,8 | **5.26** |
|  | P31120 | glmM | Phosphoglucosamine mutase  Escherichia coli - [GLMM_ECOLI] | 2,8 | **5.71** |
|  | P75823 | ltaE | Low specificity L-threonine aldolase  Escherichia coli - [LTAE_ECOLI] | 2,8 | **5.81** |
|  | P02925 | rbsB | D-ribose-binding periplasmic protein  Escherichia coli - [RBSB_ECOLI] | 2,8 | **5.99** |
|  | P21179 | katE | Catalase HPII  Escherichia coli - [CATE_ECOLI] | 2,7 | **5.54** |
|  | P0ABA4 | atpH | ATP synthase delta chain  Escherichia coli - [ATPD_ECOLI] | 2,6 | **4.94** |
| **Decreased Interaction Potential (DIP)** | P0A7V8 | rpsD | 30S ribosomal protein S4. Escherichia coli - [RS4_ECOLI] | 0,1 | **10.5** |
|  | P0A7S9 | rpsM | 30S ribosomal protein S13. Escherichia coli - [RS13_ECOLI] | 0,1 | **10.78** |
|  | P0A9G6 | aceA | Isocitrate lyase Escherichia coli - [ACEA_ECOLI] | 0,2 | **5.16** |
|  | P0A9M8 | pta | Phosphate acetyltransferase Escherichia coli - [PTA_ECOLI] | 0,2 | **5.28** |
|  | P02359 | rpsG | 30S ribosomal protein S7.  Escherichia coli - [RS7_ECOLI] | 0,2 | **10.37** |
|  | P60422 | rplB | 50S ribosomal protein L2.  Escherichia coli - [RL2_ECOLI] | 0,2 | **10.93** |
|  | P0A7V3 | rpsC | 30S ribosomal protein S3.  Escherichia coli - [RS3_ECOLI] | 0,3 | **10.27** |
|  | P0A7R9 | rpsK | 30S ribosomal protein S11.  Escherichia coli - [RS11_ECOLI] | 0,4 | **11.33** |
|  | P23836 | phoP | Transcriptional regulatory protein phoP. Escherichia coli - [PHOP_ECOLI] | 0,4 | **5.1** |
|  | P60438 | rplC | 50S ribosomal protein L3.  Escherichia coli - [RL3_ECOLI] | 0,4 | **9.9** |

**Table S5.**

| **Protein name** | **Number of paralells** | **Mean** | **Standard Deviation** | **Standard  Error of Mean** |
| --- | --- | --- | --- | --- |
| GST | 24 | 0,389 | 0,127 | 0,026 |
| CAST | 18 | 0,387 | 0,064 | 0,015 |
| ERD14 wt | 42 | 0,739 | 0,128 | 0,020 |
| Full-Scr | 21 | 0,395 | 0,152 | 0,033 |
