# Supplementary figures and images for: Interplay of Structural Disorder and Short Binding Elements in the Cellular Chaperone Function of Plant Dehydrin ERD14
Source: Cells. 2020 Aug 7;9(8):1856. doi: 10.3390/cells9081856 (PMC7465474; doi:10.3390/cells9081856)

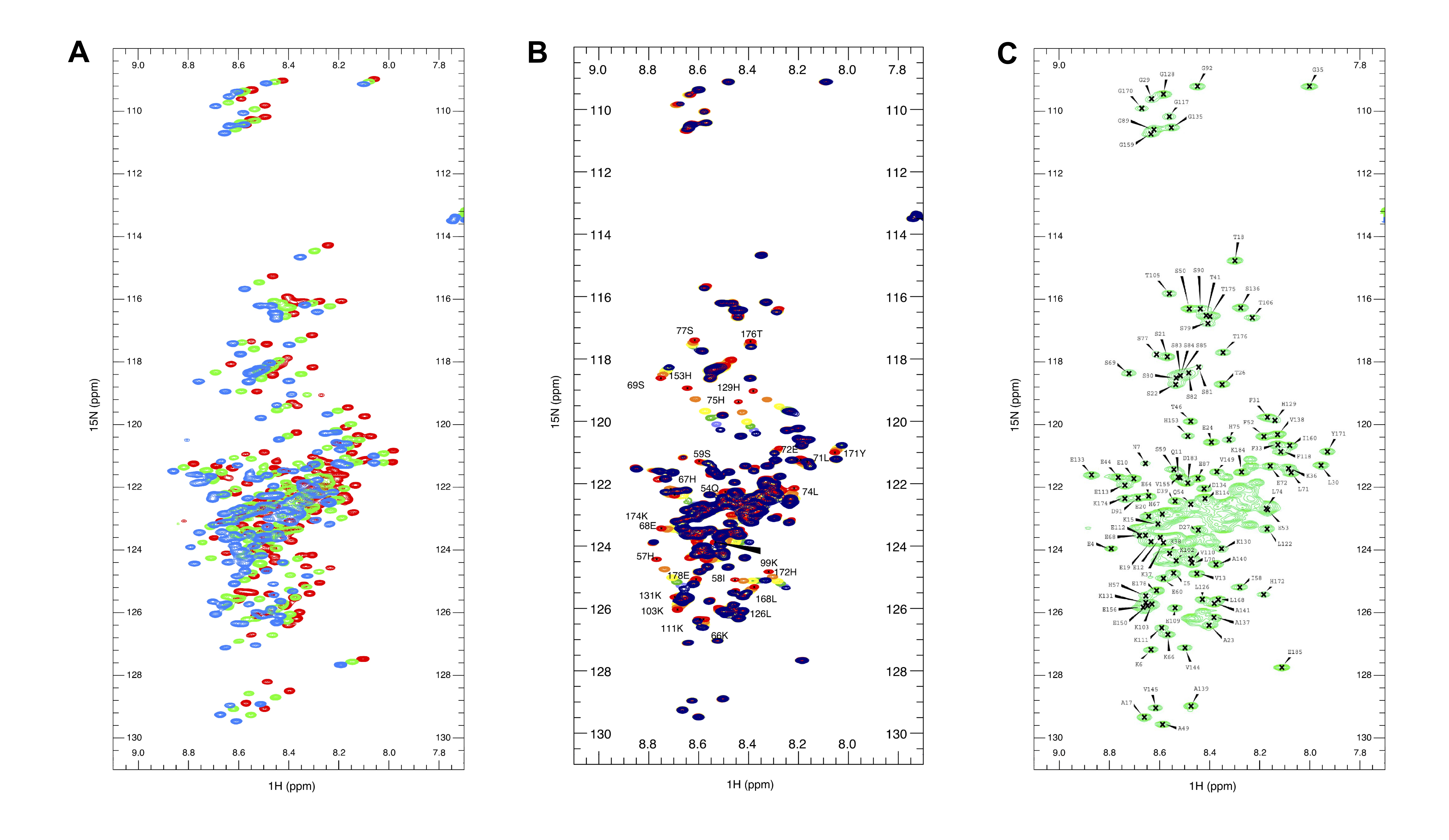

Supplement: Supplementary file 1 [file cells-09-01856-s001.zip › Figure S1_final.jpg]

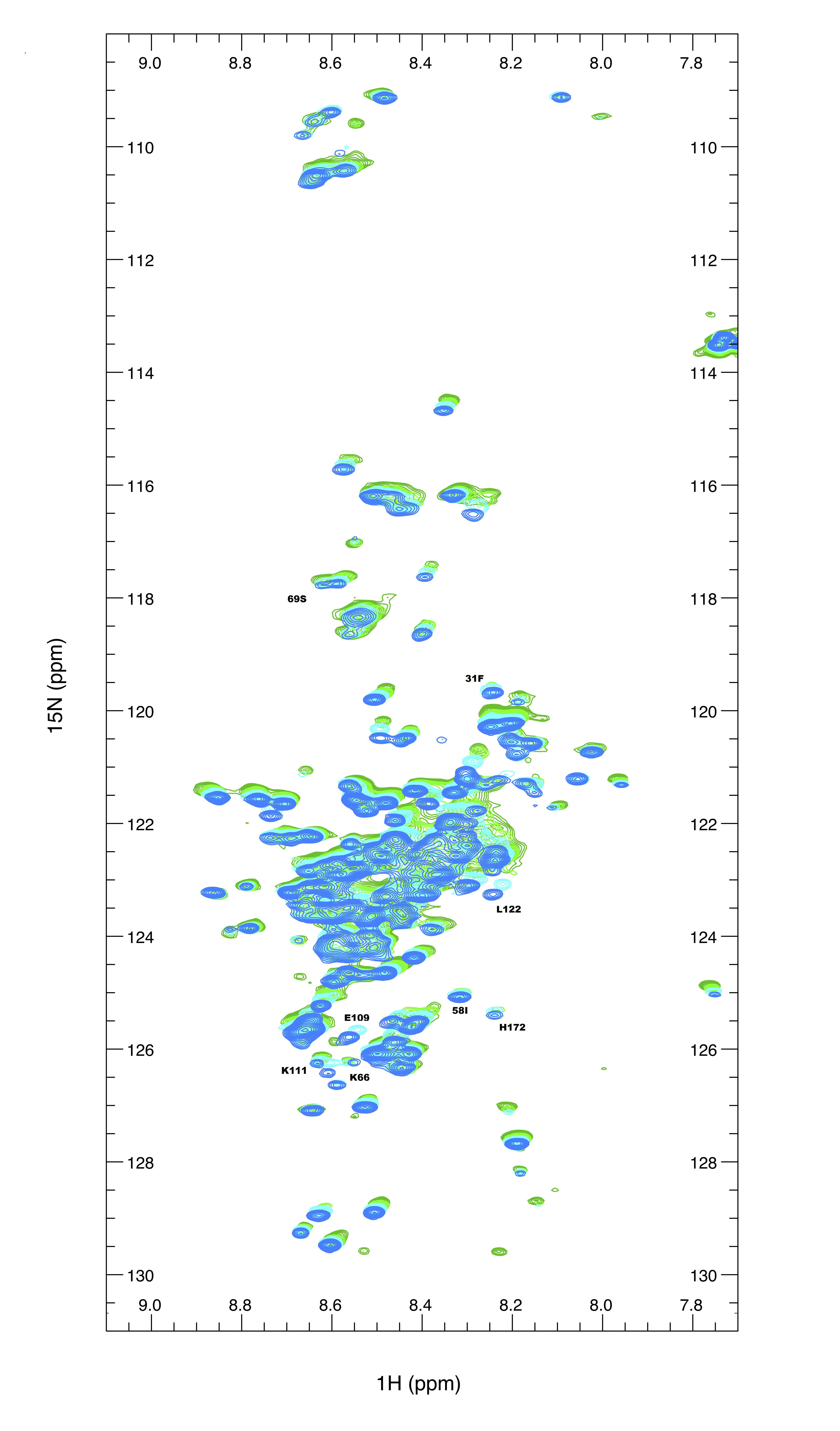

Supplement: Supplementary file 1 [file cells-09-01856-s001.zip › Figure S2_final.jpg]

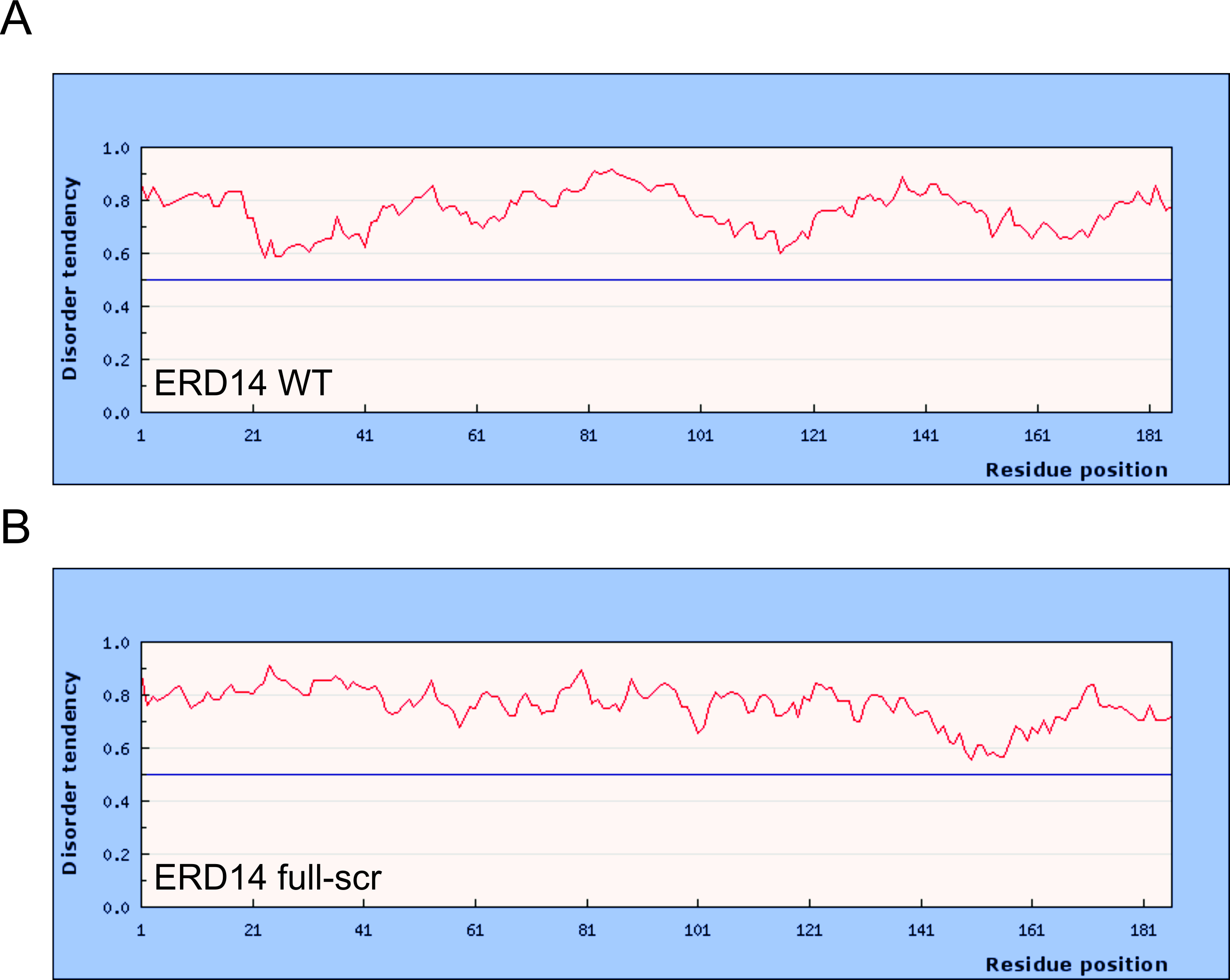

Supplement: Supplementary file 1 [file cells-09-01856-s001.zip › Figure S3_final.jpg]

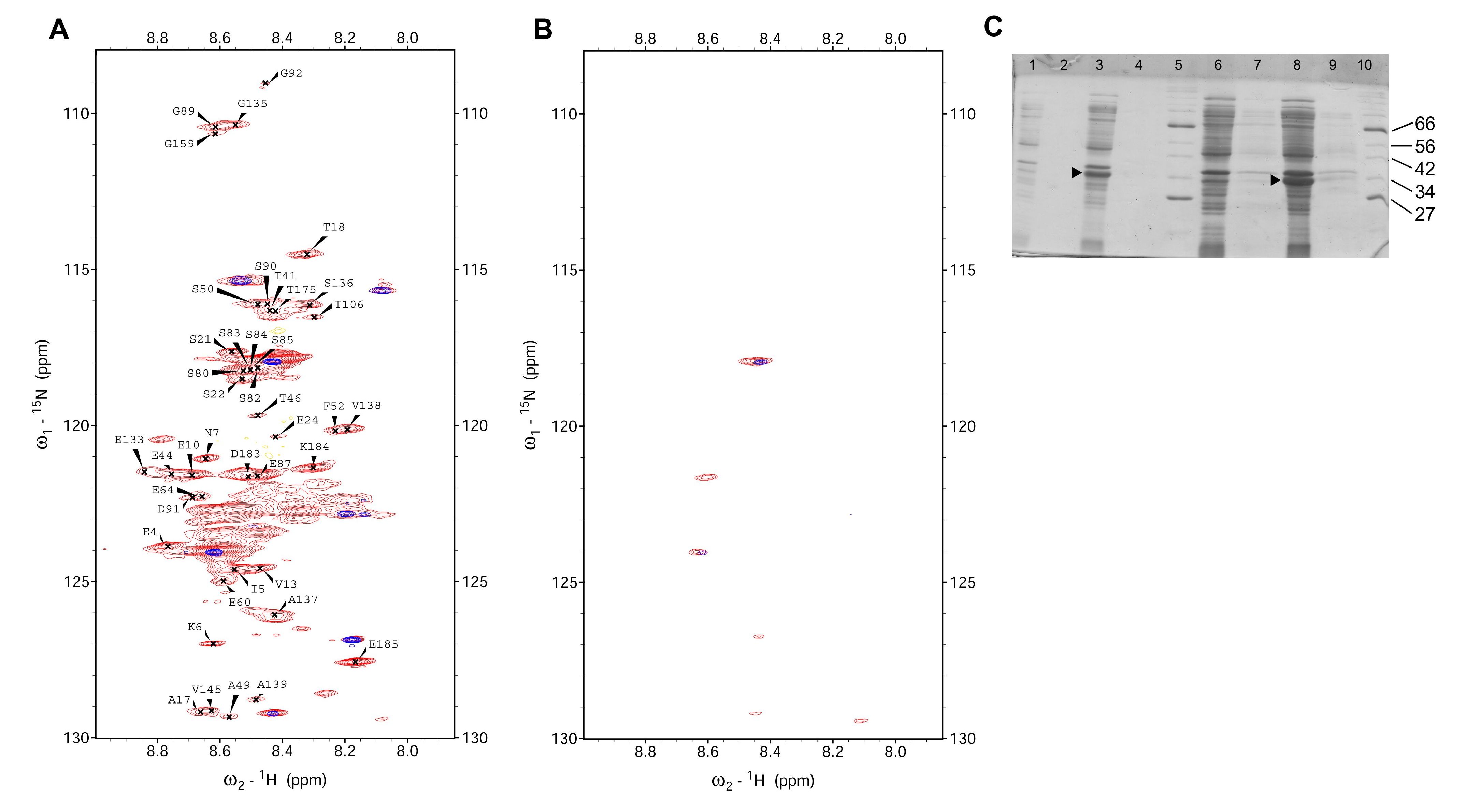

Supplement: Supplementary file 1 [file cells-09-01856-s001.zip › Figure S4_final.jpg]

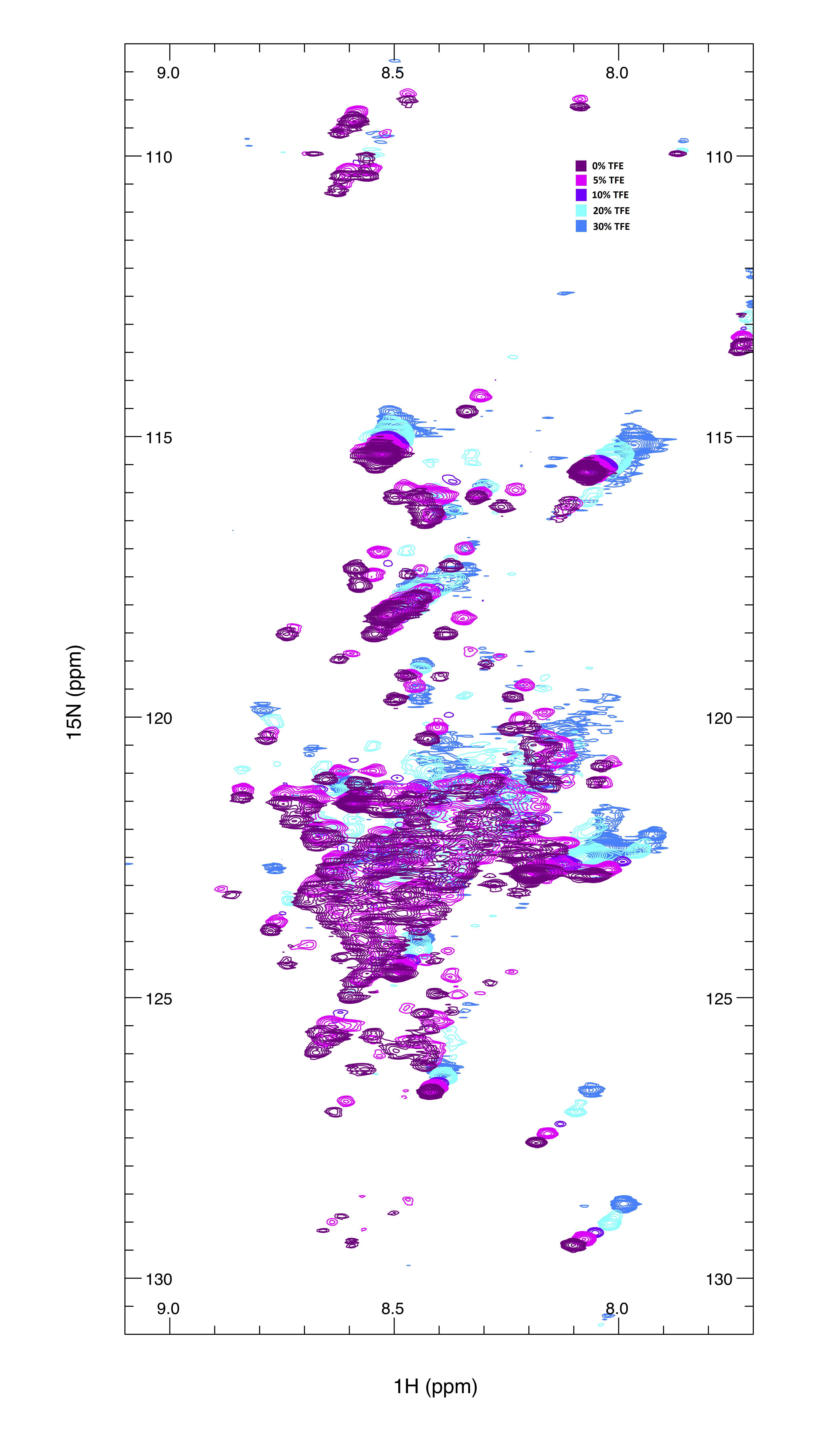

Supplement: Supplementary file 1 [file cells-09-01856-s001.zip › Figure S5.jpg]

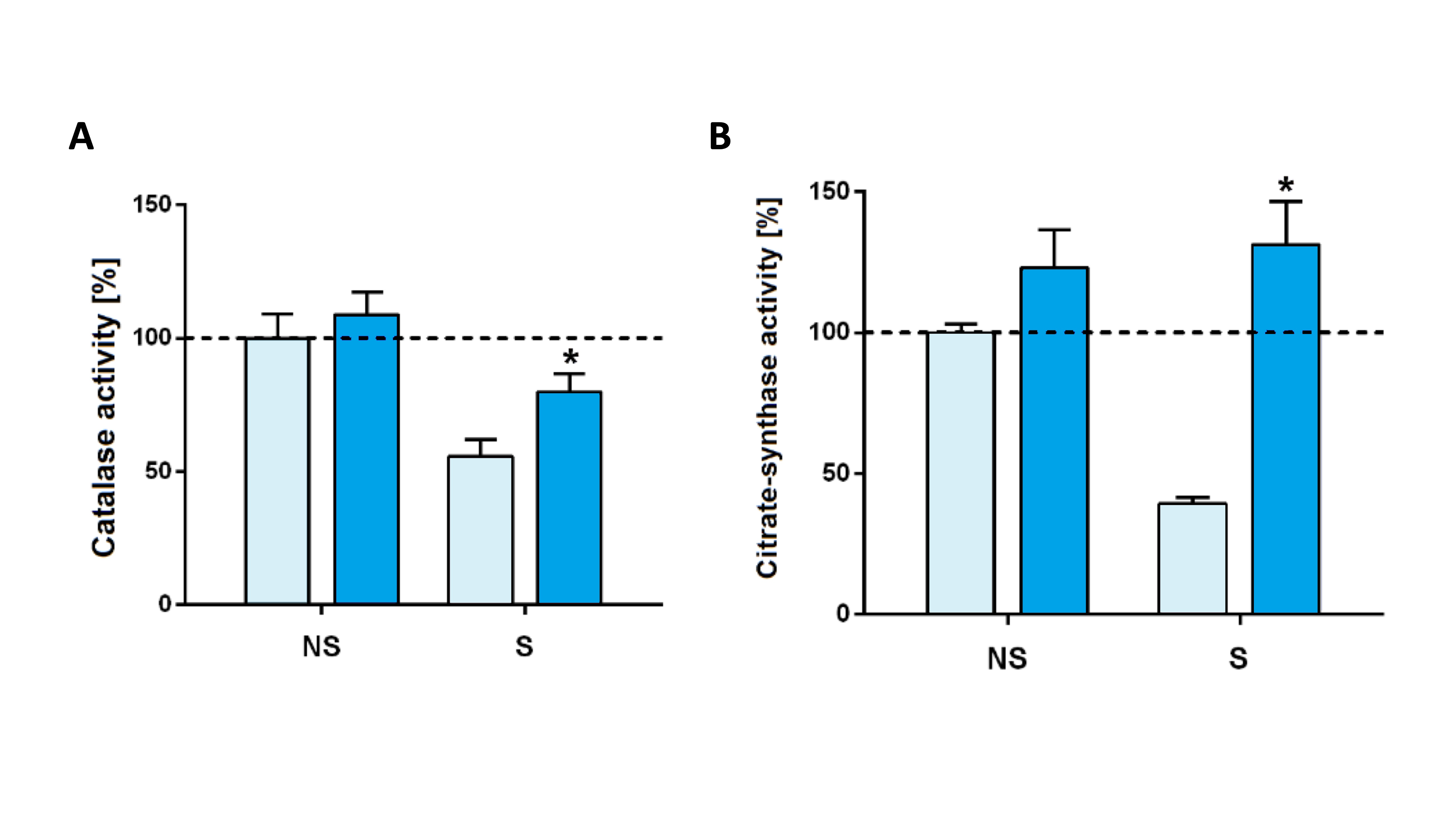

Supplement: Supplementary file 1 [file cells-09-01856-s001.zip › Figure S6.tiff]

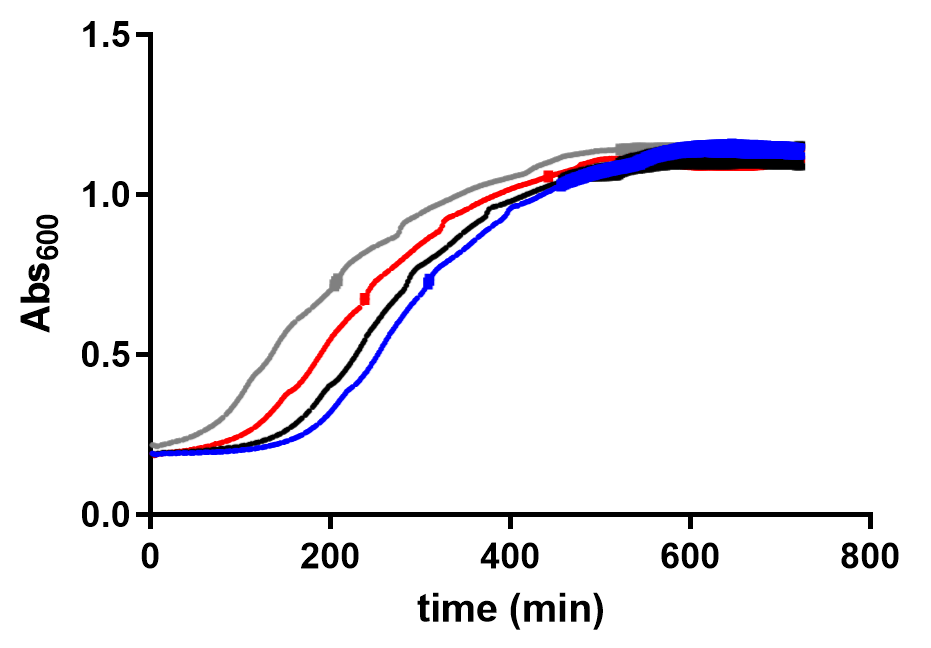

Supplement: Supplementary file 1 [file cells-09-01856-s001.zip › Figure S7.tif]
